# Applying Bayesian Multivariable Mendelian Randomisation to Prioritise Candidate Causal Traits From High‐Dimensional Data: Illustration From Estimation of the Effect of Maternal Metabolites on Offspring Birthweight

**DOI:** 10.1002/gepi.70043

**Published:** 2026-06-30

**Authors:** Ciarrah‐Jane Barry, Verena Zuber, Deborah A. Lawlor, Maria Carolina Borges, Eleanor Sanderson, Chin Yang Shapland

**Affiliations:** ^1^ MRC Integrative Epidemiology Unit University of Bristol Bristol UK; ^2^ Population Health Sciences, Bristol Medical School University of Bristol Bristol UK; ^3^ MRC Biostatistics Unit, School of Clinical Medicine University of Cambridge Cambridge UK; ^4^ Department of Epidemiology and Biostatistics, School of Public Health Imperial College London London UK; ^5^ NIHR Bristol Biomedical Research Centre Bristol UK

**Keywords:** Bayesian model averaging, birthweight, maternal, metabolites, multivariable Mendelian randomisation, offspring

## Abstract

Mendelian randomisation (MR) is an approach to causal inference that uses genetic variants to infer whether or not a causal effect exists, unbiased by unobserved confounding. MR estimation usually considers the effect of a single exposure on an outcome; it has recently been extended to explore potential effects of multiple exposures using multivariable MR (MVMR). Existing MVMR models are restricted to a few exposure traits in a single estimation, particularly if those traits are highly correlated. However, for many relationships of interest there are many highly correlated exposures which may have a causal effect on the outcome. MVMR Bayesian model averaging (MVMR‐BMA) provides a hypothesis‐free exposure selection approach with many correlated exposures. Although potentially very powerful, BMA approaches to estimation are not commonly applied in epidemiological studies. Here we describe the application of MVMR‐BMA to the selection of maternal metabolites that are causal for offspring birthweight. We describe the inputs and outputs of the model in detail and discuss the appropriate sensitivity analyses, illustrating these with our application. Through this, we hope to provide a guide to help other researchers, who are potentially unfamiliar with the terminology of Bayesian analysis, but would like to apply the method to their data.

## 1 Introduction

Confounding in epidemiological studies can bias observed associations between proposed exposures and outcomes, meaning that determining the presence of a causal effect of the exposure on the outcome is challenging. Mendelian randomisation (MR) is an approach that uses genetic variants to infer whether a causal effect exists, unbiased by unobserved confounding. MR is commonly implemented as an instrumental variable analysis, using genetic variants as instruments to estimate causal effects (Davey Smith and Hemani 2014; Davey Smith and Ebrahim 2003; Lawlor et al. 2008; Sanderson et al. 2022).

MR can be extended to include multiple, potentially correlated, exposures in a single estimation through multivariable MR (MVMR) estimation (Sanderson et al. 2021; Burgess and Thompson 2015). MVMR allows for potential pleiotropic pathways from the genetic variants used as instruments to the outcome to be accounted for. Researchers are often interested in relationships where there are many highly correlated potential exposures. However, as the number of exposures in the model increases, MVMR is less reliably able to estimate causal effects, particularly within large sets of highly correlated exposures (Karageorgiou et al. 2023). In this paper, we will consider a specific example, the effect of maternal metabolites on offspring birthweight. There are numerous potential maternal metabolites, which are highly correlated and each of which may have a causal effect on offspring birthweight, such as glucose, amino acids and lipids (Lawlor et al. 2012; Kadakia et al. 2019; Kulkarni et al. 2013; Barry et al. 2022). Therefore, it is necessary to take an approach to select a subset of the metabolites through exposure selection.

One approach to exposure selection is to select only traits that show evidence of an effect in a univariable model, forward to the MVMR estimation. We have previously applied this approach to estimate the effect of maternal metabolites on offspring birthweight (Barry et al. 2022). An alternative method, which enables exposure selection in settings with large numbers of highly correlated potential exposures, is MVMR Bayesian model averaging (MVMR‐BMA) (Zuber, Colijn, et al. 2020). This method uses a hypothesis‐free exposure selection approach, avoiding the researcher bias that arises when analysts select potential exposures from a larger set themselves.

MVMR‐BMA has been used in a number of previous analyses and prioritised biologically plausible exposures from several hypothesised and correlated exposures. Lipid metabolism is known to play a major role in the risk of cardiovascular diseases, such as coronary artery disease (CAD) and peripheral artery disease (PAD) (Duran et al. 2020; Aday et al. 2018); however, identifying causal effects from specific lipid or lipoprotein components is challenging given their high‐dimensional, correlated structure (Duran et al. 2020; Aday et al. 2018). MVMR‐BMA has supported apolipoprotein B being the most likely causal risk factor for PAD, prioritising apolipoprotein B over high‐density lipoprotein cholesterol, low‐density lipoprotein cholesterol, triglycerides and apolipoprotein A (Levin et al. 2021). A different study used MVMR‐BMA to prioritise 30 lipoprotein measures and metabolites as causal risk factors for CAD. This study identified that apolipoprotein B had the highest probability of being a causal risk factor for CAD and that the most likely model included only apolipoprotein B. MVMR‐BMA has also been applied to prioritise exposures for a range of other outcomes and has identified; fasting insulin, type 2 diabetes and hip circumference as most likely causal risk factors for osteoporosis (Zhang et al. 2021), haemoglobin for venous thromboembolism (Luo et al. 2020), extra‐large HDL and total cholesterol for age‐related macular degeneration (Zuber, Colijn, et al. 2020).

The results identified above highlight the potential use of MVMR‐BMA to identify and priortise potential risk factors when the large number of potential exposures limits the ability to use coventional epidemiological or MR approaches. However, Bayesian approaches to estimation may not be familiar to many epidemiological researchers who may wish to apply this method in their research. In this paper, we provide a clear and accessible explanation of the method, beginning with an overview of Bayesian model averaging and the MVMR‐BMA framework to explain how the method should be implemented and interpreted. We describe the user‐specified inputs required and how these may influence the analysis. We describe how to interpret the results obtained, alongside highlighting the recommended robustness and sensitivity analyses. We illustrate this guide through an application of MVMR‐BMA to estimate the effect of a range of maternal metabolites on offspring birthweight, extending and validating the analysis we have published previously (Barry et al. 2022). We highlight the features that indicate when the method can reliably distinguish between models, demonstrating what to look for in practice. We reflect on the characteristics of the data that are required for estimation with MVMR‐BMA, which are likely to apply to many MVMR methods applied to similar data structures. Through this, we aim to provide an accessible tutorial for researchers seeking to understand and implement MVMR‐BMA, whilst highlighting considerations and potential pitfalls that may occur in practice.

### 1.1 MVMR‐BMA

**Figure 1:**
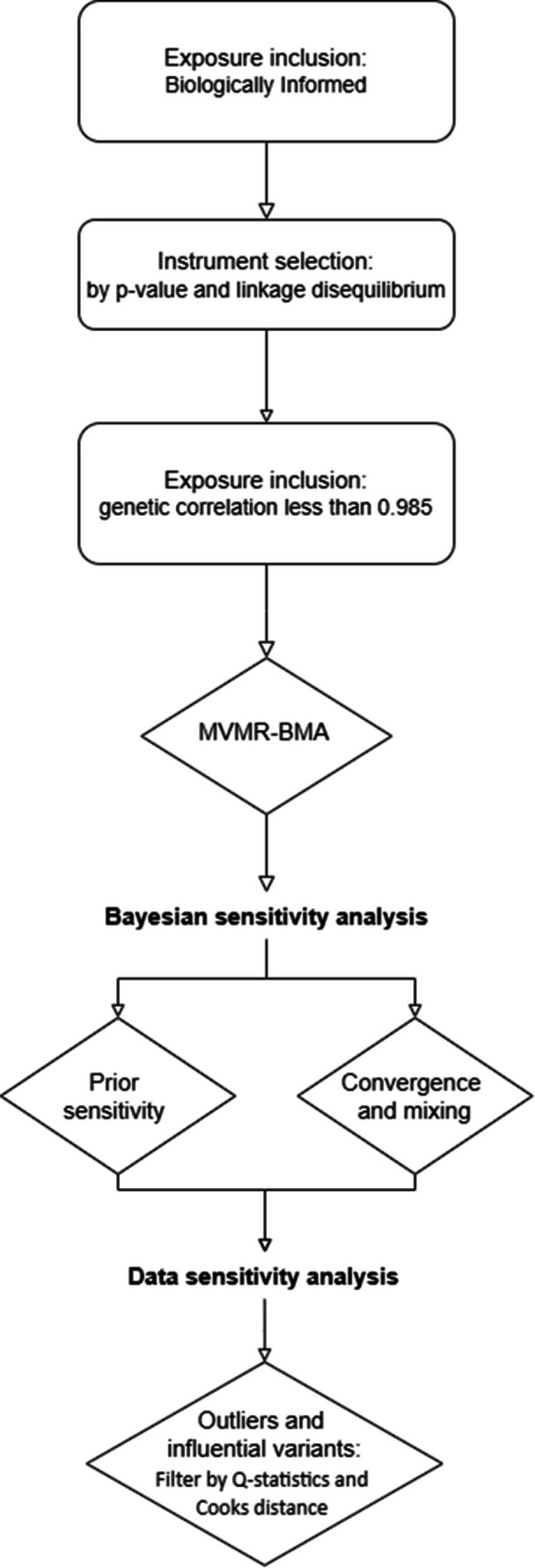
Summary of recommended steps to run MVMR‐BMA analysis.

In this section, we explain MVMR‐BMA, the inputs required, and the interpretation of the outputs with reference to our application throughout. Figure 1 gives the summary of suggested steps when running MVMR‐BMA.

Bayesian model averaging is a methodological approach to estimation which incorporates uncertainty in the model specification, thus reflecting that the true causal model is a priori unknown. Within a Bayesian model averaging approach an algorithm searches over a range of different models to estimate the probability with which each is the likely true model and incorporates model uncertainty in the effect estimate. The different models considered could be different exposures, but could also be various specifications of other aspects of the model, such as whether a relationship is linear or whether a non‐linear model should be used. It is a hypothesis‐free approach that avoids the need for the researcher to specify a single assumed ‘true’ model prior to estimation.

MVMR‐BMA uses a Bayesian model averaging approach to select which exposures out of a larger set of, potentially correlated, exposures are causal exposures for the outcome, within an MR framework (Zuber, Colijn, et al. 2020). For each model, the effects are estimated using conventional Bayesian MVMR approaches based on conjugate Normal priors and thus are in spirit close to Ridge regression. The models are estimated using a pre‐specified set of SNPs each of which are associated with at least one of the potential exposures, using the same specifications for association as would be applied in a `conventional MR estimation (Sanderson et al. 2022, 2019; Burgess and Thompson 2015). MVMR‐BMA considers all possible combinations of exposures from the initial set (up to a pre‐defined maximum included in any one model) and incorporates model uncertainty through calculating a weighted average of the effect estimate from each model (Hoeting et al. 1999). Every model considered is given a model‐specific posterior probability (PP) of being the true causal model. The PP is the probability of how likely the model is to be the true causal model, enabling comparison of the different models considered by the method. The PP is estimated based on a combination of prior probability for that particular model and the Bayes factor calculated from the data. Bayes factor is a commonly used statistic for Bayesian model comparison, which compares the likelihood of one model relative to another (Zuber, Colijn, et al. 2020).

The BMA algorithm runs for many iterations, selecting a different model each time but returning to some models more often due to a relatively high likelihood of being the true model calculated by the estimation (and/or high prior probability specified by the researcher of being the true model). While for a smaller number of exposures, the traditional BMA (Hoeting et al. 1999) can exhaustively evaluate all models, that is, all possible combinations of exposures, this becomes computationally infeasible with standard desktop computing resources when the number of exposures approximately exceeds 20 (total number of possible models is 2^20^). Zuber et al. (Zuber, Colijn, et al. 2020). have used shotgun stochastic search to reduce the burden. This allows the method to be applied to sets of exposures that are much larger than 20 without excessive computational burden. The Supplementary Notes and Supporting Information S1: Figure S1 compares runtime for different algorithm using simulated data.

For each individual exposure, MVMR‐BMA aggregates the evidence across models in which the exposure is included and calculates the marginal‐inclusion probability (MIP) and the model‐average direct causal effect. The MIP for each exposure is an estimate of how likely that exposure is to have a causal effect on the outcome and is calculated as the sum of PP across all models that contain the corresponding exposure. The model‐averaged direct causal effect of the exposure on the outcome is the effect estimate for each exposure, averaged across the models that include the corresponding exposure (Zuber, Colijn, et al. 2020).

### 1.2 Model Input Considerations

#### 1.2.1 Exposure Inclusion

As the MVMR‐BMA algorithm considers all possible combinations of exposures there are practical limits to the number of exposures that can be included in the estimation. In this approach, the computational burden of including many traits is high; there are 2^*K*^ models for the MVMR‐BMA estimator to explore, where *K* is the number of traits included, and to estimate the posterior probability the algorithm needs to return to each model a number of times. This is due to two important aspects of Bayesian model averaging: convergence and mixing. Convergence occurs when the posterior distribution of each parameter has become stationary and reached its final value, that is, running the analysis for further iterations will not change the results obtained (Christopher et al. 2012). Mixing is the extent to which all possible models that could be considered have been considered, and is important to ensure that all exposures have a chance of obtaining an accurate probability of inclusion. When it is not possible to exhaustively evaluate all possible combinations of exposures, MVMR‐BMA implements a shotgun stochastic search. However, including further exposures will slow convergence and increase reliance on the stochastic search. One solution is to limit the number of exposures favoured by the model through a lower prior probability (described below) for each exposure. Subsequently, the algorithm favours models with fewer exposures and explores the model space for models with low numbers of exposures more thoroughly. In many applications, the most likely models are not expected to contain more than a few exposures, and this approach will lower the number of models considered without impacting the results obtained. However, it may lead to overly simplistic models being favoured, and this needs to be balanced with models with higher numbers of exposures also being considered.

Another limitation relates to the number of potential exposures is the extent to which the exposure traits are biologically and statistically overlapping and can be distinguished by the SNPs included. All MVMR approaches struggle to distinguish between noise and true causal effects when applied to many biologically or statistically overlapping traits. With any MVMR approach, the most relevant consideration is whether the genetically predicted values for each exposure, based on the genetic variants included in the estimation, are highly correlated. If the genetically predicted exposures are highly correlated it will not be possible for MVMR to distinguish those traits, and any results will be biased by (conditionally) weak instruments (Sanderson et al. 2021). This is a statistical limitation on the data that can be used with any MVMR estimation that cannot be easily avoided. As larger data sets become available, the level of correlation that can be handled within such analyses will increase.

In our example, we initially started with 249 NMR metabolic measures (details in ‘Data description’ within Supplementary Note and Barry et al. (2022)). These were therefore restricted to a smaller more feasible set by excluding multiple measures of traits that cannot be meaningfully biologically separated, leaving 48 traits (detailed explanation in Supporting Information S1: Table S1). Within the set of 48 traits, the genetic correlation between measures was calculated using the instrument‐exposure coefficient. Traits with correlation above 0.985 were excluded as they were statistically indistinguishable, removing a further two metabolites, leaving a final set of 46 traits. In this application, we applied the same threshold as in Zuber et al (Zuber, Colijn, et al. 2020). However, the precise threshold for ‘high correlation’ may vary depending on the context and specific research question being addressed by the user. When multiple highly correlated exposures are identified, we suggest retaining the exposure that is most biologically relevant or interpretable. For example, the application in Zuber et al (Zuber, Colijn, et al. 2020). included only lipoprotein measurements on total cholesterol content, triglyceride content, and particle diameter; other measures such as free cholesterol, total lipids or phospholipids were not included. Other criteria may be which exposure has the most complete genome‐wide association study (GWAS) summary data or may be supported by stronger prior evidence of relevance to the outcome. This is likely best decided by the researcher based on domain knowledge.

### 1.3 Instrument Selection

In MR, genetic variants for exposures are selected on the basis that they have a statistically strong association with the exposure(s) of interest, usually defined as being genome‐wide statistically significantly associated (*p* < 5 × 10^−8^) with the exposure in a GWAS of the exposure, pruned so only uncorrelated (R^2^ for linkage disequilibrium < 0.001) variants are retained (Sanderson et al. 2022). In MVMR, this is extended to select all genetic variants that are strongly associated with any of the exposures using the same criteria for selection as for MR (Sanderson et al. 2019).

We applied this approach to the selection of genetic variants as instruments in our estimation. We selected all variants associated with our final set of 46 traits with a *p*‐value < 5 × 10^−8^ in a GWAS of the full set of metabolites that had previously been conducted in UK Biobank (Borges et al. 2022) (summary statistics available in the IEU OpenGWAS (Bycroft et al. 2018; Elsworth et al. 2020; Hemani et al. 2018). This list was pruned to remove variants with an *R* ^2^ for linkage disequilibrium < 0.001 across the full list of variants associated with any exposure.

### 1.4 Outcome Data

For our outcome data set, we used a GWAS meta‐analysis including 210,267 European participants, combining results of 41 studies from the EGG (Early Growth Genetics) consortium and UKBB (UK Biobank), with a mean age at delivery ranging from 24.5 to 31.5 years (Warrington et al. 2019). This GWAS was adjusted for foetal genotype to avoid potential biases due to correlated with the maternal genotype (Beaumont et al. 2018). From this data set, we extracted the association between each of the SNPs identified above and offspring genotype to include in our estimation.

### 1.5 Priors

Like all Bayesian analysis, MVMR‐BMA requires prior parameters to be defined, which are probability and variance. Prior probability in this model defines the probability that any individual exposure will be a causal exposure. A smaller value informs the algorithm to favour models with fewer exposures, and a larger value to favour models with many exposures. Zuber et al. (Zuber, Colijn, et al. 2020) recommend specifying the probability based on the expected model size (i.e., probability times number of exposure). The causal effect estimate follows a normal prior centred at zero with a user defined variance of *σ* ^2^. This specification informs the method whether to favour exposures with larger or smaller causal effect estimates, with a larger *σ* ^2^ favouring exposures with larger (i.e. further away from the null) causal effects.

In the main analyses, we ran MVMR‐BMA using a prior probability of 0.1 for each of our 46 exposures being a causal exposure for birthweight, as we are expecting that the model will contain four to five metabolites (= 0.1 × 46). The prior variance was set to 0.5 for each metabolite, as we would like to allow for stronger causal effects. We also ran sensitivity analyses of different choices of prior probability and variance.

## 2 Main Results

Tables 1 and 2 give the top five models and exposures, respectively, from our estimation, full results are given in Supporting Information S1: Tables S2 and S3. The top model, that is, model with the highest PP, selected by MVMR‐BMA contained glutamine and glucose, with a model PP of 0.377 (Table 1). The second model contained only glucose with a model PP of 0.311. After these top models, the PP of subsequent models was much lower, suggesting that the method strongly weights the models with glutamine and glucose or only glucose as being the most likely.

**Table 1:** The top five ranked models for birthweight according to their posterior probabilities (PP) and model‐specific causal estimates.

Model	PP	Model‐specific causal estimates
*Main analysis (L* = *598, K* = *46)*		
Glutamine, glucose	0.377	0.058, 0.237
Glucose	0.311	0.244
Alanine, glutamine, glucose	0.051	0.067, 0.058, 0.221
Alanine, Glucose	0.039	0.066, 0.228
Glutamine, glucose, lactate	0.021	0.060, 0.254, 0.076
*Robustness: Influential and outlying variants removed (L* = *595, K* = *46)*		
Glucose	0.453	0.218
Glucose, lactate	0.180	0.241, 0.102
Glutamine, glucose	0.042	0.042, 0.214
Glucose, lactate, total lipids in small HDL	0.037	0.260, 0.127, −0.048
Glutamine, glucose, lactate	0.036	0.046, 0.238, 0.110

*Note:* Results for the top selected models by MVMR‐BMA exposure selection. *K* is number of metabolites and *L* is number of instruments. PP: Posterior Probability, Robustness: Influential variants removed shows results for analysis with outliers and influential SNPs in the model for glucose in the main analysis removed.

**Table 2:** The top five ranked exposures for birthweight according to their marginal inclusion probability (MIP).

Exposure	MIP	Model averaged causal effect	Empirical *p*‐values
*Main Analysis (L* = *598, K* = *46)*			
Glucose	1.000	0.239	< 0.001
Glutamine	0.551	0.032	0.005
Alanine	0.117	0.008	0.037
Lactate	0.041	0.003	0.274
Total lipids in small HDL	0.021	−0.001	0.249
*Robustness: Influential and outlying variants removed (L* = *595, K* = *46)*			
Glucose	1.000	0.227	—
Lactate	0.328	0.035	—
Glutamine	0.108	0.005	—
Total lipids in small HDL	0.070	−0.003	—
Acetate	0.035	−0.002	—

*Note:* Results for the top ranked exposures by MVMR‐BMA exposure selection. K is number of metabolites and L is number of instruments. MIP: Marginal inclusion probability, Model averaged causal effect: weighted average causal effect across all models in which this exposure is selected, empirical *p*‐value: give an indication of how extreme an estimate is relative to a null expectation via permutation. Robustness: Influential variants removed shows results for analysis with outliers and influential SNPs in the model for glucose in the main analysis removed.

The MIP estimates the probability of a particular exposure being included in the final model, shown in Table 2. The MIP for glucose was 1.00 showing that it was selected by all likely models. Glutamine was the exposure with the second highest MIP with a value of 0.551, followed by alanine with a MIP of 0.117. These results reflect the weighting given to the models when including each of these traits.

In addition to the MIP for each exposure, empirical *p*‐values can be calculated to provide evidence on whether a particular exposure has a causal effect on the outcome, see Supplementary note ‘Empirical *p*‐values’. A small *p*‐value is supportive of rejecting a null hypothesis, that the exposure does not affect the outcome, in favour of an alternative hypothesis, that there is a causal effect. These empirical *p*‐values are calculated using a permutation approach with adjustment for multiple testing (Tang et al. 2022; Levin et al. 2021). The calculation of the empirical p‐values requires the MR‐BMA algorithm to be repeated at least 1k or 10k times to return stable results. We therefore restricted the generation of the permutation p‐values to an exhaustive search over a search space of all models, including up to four exposures for our primary analysis.

MVMR‐BMA reports two sets of causal effect estimates; the model‐specific causal effect estimates, and the model‐averaged causal effect estimates. The model‐specific causal effects are the direct effect estimated for each exposure in the model specified, conditional on the other exposures included in the model. Where some exposures partially mediate the effect of another exposure, these estimated effects could differ across different models. Model‐averaged causal effects are also reported, which give the average causal effect estimated for each exposure, averaged across all models in which that exposure is included. The model‐specific and model‐averaged causal effect estimates returned by the MVMR‐BMA are attenuated towards the null where there is a true causal effect. This occurs because the models prefer a reduction in the variance of the estimates over a reduction in bias. The conservative bias towards the null has been demonstrated in simulations (Zuber, Colijn, et al. 2020). In addition, no measure of the uncertainty around these effect estimates is available. Therefore, the causal effect estimates should not be interpreted as unbiased estimates of the causal effect of each exposure on the outcome, but the interpretation of the results should be focused on exposure selection rather than effect estimation.

## 3 Bayesian Sensitivity Analysis

Sensitivity analyses to check the model should be applied to any MVMR‐BMA (or indeed any Bayesian approach), as large variation of the results across robustness checks indicates that initial specification for the analysis is influencing the results obtained, and needs to be considered carefully.

### 3.1 Priors

It is possible that the chosen prior will have a substantial influence on the result obtained from the MVMR‐BMA, and therefore the estimation should be run for a range of priors. If a model is consistently selected across priors, it can be considered to be robust as the prior itself is not determining the selected model. We therefore tested a range of values for prior probability and prior variance for our model. All the results are in Supporting Information S1: Table S4 and S5. For prior probability, we considered values of 0.01, 0.05 and 0.20, and for prior variance, we considered values of 0.30, 0.50 and 0.70. As expected, with a smaller prior probability, the model with fewer exposures (only glucose) is favoured over the model with more exposures (glutamine and glucose). When a larger prior probability is used, the model with both glutamine and glucose is favoured over just glucose (Supporting Information S1: Table S4). A prior variance of 0.7 led the BMA to select larger values for the causal effect estimate, in our case glucose has the largest effect (model‐specific causal effect of 0.24 for the model only including glucose) compared to other metabolites, and therefore the model with glucose only becomes most favoured by BMA PP changed from 0.311 to 0.41 (Supporting Information S1: Table S5a). However, the model with glutamine and glucose and the model with glucose are consistently favoured from the MVMR‐BMA procedure. Therefore our BMA analyses are robust to differing values of prior variance and prior probability.

### 3.2 Convergence and Mixing

Initially our MVMR‐BMA algorithm was run using 100,000 iterations throughout, however we additionally re‐ran the algorithm with 500,000 iterations to check that the algorithm was being run for sufficient iterations. We obtained identical results to the main analysis (Supporting Information S1: Table S6). We conclude 100,000 iterations were sufficient for convergence and mixing. Thus we used 100,000 iterations for all of the sensitivity analyses.

## 4 Data Sensitivity Analyses

We applied a number of sensitivity analyses to our estimation to identify whether the results were being driven by characteristics of the data we were using. These sensitivity analyses are standard for any MR estimation to understand how the results depend on the particular data set used.

### 4.1 Outliers and Influential Variants

MR estimation relies on a set of IV assumptions for the estimates obtained to be interpreted as causal effects of the exposure(s) on the outcome (Sanderson et al. 2022). One assumption is that the SNPs used as instruments only affect the outcome through the exposure and not by any other pathway. If the majority of the SNPs used in the estimation are valid (in the sense that they satisfy this condition), then SNPs that violate the assumption will give individual effect estimates that differ from those for the larger set of SNPs. These SNPs will be outliers if the individual SNP level estimates are considered, and will make the total heterogeneity in the effect estimates across all SNPs larger. One way heterogeneity in the model can be measured is through a *Q*‐statistic. The appropriate critical value for this test can be obtained from a $\chi^{2}$ distribution with degrees of freedom equal to the number of SNPs minus the number of exposures. A statistically significant value for the Q‐statistic (i.e., larger than the relevant critical value 0) indicates heterogeneity in the model is higher than would be expected purely by statistical noise (Bowden et al. 2018).

Similarly, particularly influential SNPs that have more influence on the results obtained, may make the results from the analysis less robust if those SNPs are the ones that violate the IV assumptions. Cook's distance is a measure of the influence of any individual data point (in this case, SNP, as we are conducting analysis with summary statistics) on the estimates obtained from a regression analysis. In this setting, each SNP is a data point, and the IVW estimation is the regression in question.

To assess the influence of outliers and influential SNPs in MVMR‐BMA we re‐ran the analysis excluding any SNPs that were found to be outliers or highly influential in the top model (including glutamine and glucose). SNPs were defined as outliers where the individual SNP‐level contribution to the overall *Q* statistic was greater than the relevant critical value (here 22.78). Highly influential SNPs were defined to be those exceeding the Cook's distance threshold for the IVW estimates (here 0.987). Note: these thresholds are dependent on the number of SNPs and so will differ across applications. A total of three outlying and zero highly influential SNPs were removed based on these criteria. *Q*‐statistics and Cook's distance are reported in Supporting Information S1: Tables S7 and S8 and Figure S2.

This exclusion changed the top models selected, the model with the highest PP was now the glucose‐only model, while the glucose and lactate model ranked second, which was previously not in the top five. Correspondingly, the MIP for lactate increased, and the MIP for glutamine decreased. The SNPs removed were rs1801133, rs2168101 and rs7137828. Outlying SNPs should be removed from any MR analysis with caution. A SNP being outlying does not necessarily mean that it is pleiotropic, and it is possible that outlying SNPs are the SNPs that give the true causal effect. Investigation of the identified outlying SNPs showed that rs2168101 is strongly associated (*p* < 5 × 10^−8^) with 5 of the 46 metabolites, including both glutamine (*p* = 7.6 × 10^−48^) and alanine (*p* = 9.0 × 10^−31^). rs1801133 is only associated with glycine (*p* = 1.8 × 10^−8^). rs7137828 is strongly associated with 15 traits (*p* < 5 × 10^−8^) including lactate (*p* = 6.8 × 10^−24^). It is not possible to determine from this data alone whether the removal of these SNPs has removed pleiotropic effects in the estimation or if the removal of these SNPs has changed the results obtained because the ability of the SNPs used as instruments to predict glutamine, alanine and lactate has changed between the two models. However, the result that glucose is the top exposure in each estimation adds strength to the conclusion that glucose is an important determinant of birthweight based on genetic evidence.

### 4.2 Exposure Inclusion

Two exposures, total lipids in very large VLDL and total lipids in small LDL, were excluded from the main analysis due to their high genetic correlation with total lipids in large VLDL and total lipids in medium LDL, respectively. Therefore, as sensitivity, we excluded the total lipids in large VLDL and total lipids in medium LDL instead, to include their correlated counterpart. Similar results to the main analysis were obtained (Supporting Information S1: Table S11).

## 5 Discussion

Here, we have described the implementation of MVMR‐BMA and discussed the range of robustness and sensitivity analyses that should be conducted as part of a MVMR‐BMA estimation. In our application, we found maternal glucose had a high probability of having a causal effect on offspring birthweight. This result is not surprising as glucose is a well‐established maternal metabolite driving offspring birthweight as reported by both RCTs (Farrar et al. 2017) and MR studies (Barry et al. 2022; Tyrrell et al. 2016; Chen et al. 2020). However, other exposures selected by the model were sensitive to the exclusion of only three outlying SNPs, out of 598, indicating the final specification of the model selected was sensitive to the data used. In this final section, we discuss the characteristics of the data set that may have determined these results in the context of our applied example, alongside considerations for the interpretation of robustness and sensitivity analyses that assessed the performance of the MVMR‐BMA procedure. Additionally, we highlight the types of data features and challenges that may affect model performance. As such, we hope to provide a guide to future researchers applying the method of what data characteristics will make applying this method, and indeed any multivariable MR method, challenging and how to identify those issues.

### 5.1 Instrument Strength in MVMR‐BMA

A key assumption for MR and MVMR estimation is that the exposure(s) can be strongly predicted by the genetic variants used as instruments in the relevant population for the estimation. *F*‐statistics in for the association between the instruments and the exposure can be used to test the strength of the instruments in an estimation (Sanderson et al. 2022). A value of 10 or higher is often used as an indicator that there is unlikely to be substantial weak instrument bias in the estimation, based on an approximation of the critical value for a standard test for weak instruments (Stock and Yogo 2005). This relies on the assumption that the data where the exposure is measured in is the relevant population for the outcome, and so in the example here, assumes that the association between the genetic variants and the metabolites of interest is the same in pregnant women (the relevant population for outcome data) as it is in the general population (in which our exposure data was measured).

MVMR‐BMA includes all SNPs associated with any candidate trait in each model considered. If only very few of the SNPs included are associated with a trait that has a causal effect on the outcome, then most of these SNPs will show evidence of no causal effect on the outcome. This may lead the MVMR‐BMA algorithm to struggle to distinguish between noise, driven by the many SNPs associated with non‐causal traits, and the true effect, which is seen only for a few SNPs. In our application, 17 of the 598 SNPs included in our main analysis are associated with glucose at genome‐wide significance level. For lactate, which was only selected by our sensitivity analyses, 7 of the 598 SNPs were strongly associated (Supporting Information S1: Table S9). Weak instruments in MVMR estimation can bias the causal effects either towards or away from the null, however, they also lead to higher uncertainty around the estimates obtained. This can lead to lower support for a model being the true causal model, down weighting the PP given to the model in a MVMR‐BMA approach.

The issue of weak instruments is not specific to MVMR‐BMA but will appear, in slightly different forms, in any MVMR analyses attempting to estimate multiple traits when only a few SNPs are associated with an exposure of interest (Sanderson et al. 2021). We calculated conditional *F*‐statistics, see Supporting materials ‘Conditional F‐statistics’, for each of the top models selected by the MVMR‐BMA to assess the influence of weak instruments on model selection (Sanderson et al. 2021). Conditional F‐statistics for each exposure ranged from 8.3 to 83.6 (Supporting Information S1: Table S10) when only SNPs associated with the selected exposures were considered. However, when all SNPs are included in the estimation, as is the case in MVMR‐BMA, these ranges dropped to 2.6 to 12.5. This difference occurred because the *F*‐statistic is calculated as an average across all genetic variants used as instrumental variables in the estimation. Glucose had a conditional F‐statistic of approximately 4.6–4.9, depending on exact model specification, and so the MVMR‐BMA effect estimates obtained are likely to have been biased by weak instruments. However, due to the set up of MVMR‐BMA, the causal effects are biased towards the null. Notably, lactate, which was not identified as having a high MIP in the primary analysis, had a conditional *F*‐statistic of approximately 2.7 in the models in which it was included. Effect estimates for both lactate and glucose are therefore likely biased by weak instruments, thus caution should be taken when interpreting their effect estimates (Sanderson et al. 2021). Checking the instrument strength of the exposures in the top models is important in interpreting the results from any MVMR‐BMA estimation. This issue applies similarly across MVMR estimation and currently there are limited approaches available for weak instrument robust MVMR estimation (Sanderson et al. 2021).

### 5.2 What to Look for to Check the Model Is Working

The MVMR‐BMA algorithm will always rank the models and exposures based on their PP and MIP and return a top list. This can create the appearance of having preferred one model over another when, in fact, the PP of each model is very similar. It is therefore important to check the distribution of the PP across the models. A low PP for the top model or very little variation across the top 5 or 10 models in the PP can be an indicator that there has been an error in the implementation of the algorithm or that the data being used is unsuitable for this type of estimation. In our application, the top two models, including glutamine and glucose and only glucose, respectively, had PP's of 0.377 and 0.311. In contrast, the third highest model had a PP of 0.051, showing much lower evidence of being the true causal model.

If the MVMR‐BMA does struggle to distinguish between different models, estimation could potentially be improved by restricting the set of exposures further. For example, by considering each sub‐class of metabolite separately. Although such an approach reduces the ‘hypothesis‐free’ aspect of the analysis, a ‘hypothesis‐free' approach is not possible when the genetic data is not sufficient to distinguish between noise and a true causal signal. The success of this approach will depend on the researchers' ability to reduce the exposure traits to a potentially meaningful set, where each set can be identified by the genetic instruments selected in the data.

Extensions to the MVMR‐BMA method that would also help to overcome this issue include incorporating prior probability to exposures, weak instrument robust estimation techniques, or by limiting the SNPs used for each model considered to those associated with the exposures included in that model. Neither approachhas been implemented within the algorithm so far, and each would have its own strengths and limitations. Therefore, further research is required to determine if these alterations would improve the results of the model when applied to data such as that considered here.

MVMR‐BMA reports a MIP, which is the probability that the final model includes that exposure. It can be hard to determine from a MIP whether an exposure should be selected as an exposure, as it is unclear what probability is high enough to result in inclusion of the exposure in the model. Empirical *p‐*values can be calculated for the MVMR‐BMA estimation. In connection with multiple testing procedures like false discovery rates, these empirical *p*‐values can be used to define which exposures should be selected as having a likely causal effect on the outcome (Levin et al. 2021; Zuber, Gill, et al. 2020).

## 6 Conclusion

In conclusion, MVMR‐BMA is a powerful approach that can help guide the estimation of the correct model specification when multiple, correlated exposures are available. It can be applied to much larger sets of exposures than MVMR‐IVW in a hypothesis‐free approach, avoiding the limitations of researchers only including exposures already thought to have a causal effect on the outcome in their estimation model. However, as with any estimation, when implementing MVMR‐BMA there are key limitations to be aware of. Particularly, there remains a requirement that the data is sufficient to identify the effects of the different exposures. Researchers should therefore thoroughly interrogate the results of the analysis, including through sensitivity analyses and investigation of the strength of the genetic variants to predict the exposures when interpreting the results they obtained from implementation of a MVMR‐BMA procedure.

## Author Contributions

M.C.B. conceived the idea for the paper. C.B. conducted the analysis. V.Z. and C.Y.S. conducted further analysis and code checking. C.B. wrote the first draft. All authors contributed to the interpretation of the findings. E.S. and C.Y.S wrote the second draft. All authors critically revised the paper for intellectual content and approved the final version of the manuscript.

## Ethics Statement

No ethics approval required as public summary data were used.

## Conflicts of Interest

The authors declare no conflicts of interest.

## Supporting information

Supporting File

## Data Availability

Data are publicly available at the IEU Open GWAS project (https://gwas.mrcieu.ac.uk/). Code is available at CYShapland/Maternal_metabolites_BMA_MVMR (https://github.com/CYShapland/Maternal_metabolites_BMA_MVMR).
