# Supplementary material for: Applying Bayesian Multivariable Mendelian Randomisation to Prioritise Candidate Causal Traits From High‐Dimensional Data: Illustration From Estimation of the Effect of Maternal Metabolites on Offspring Birthweight
Source: Genet Epidemiol. 2026 Jun 30;50(6):e70043. doi: 10.1002/gepi.70043 (PMC13316968; doi:10.1002/gepi.70043)
Supplement: Supplementary file 1 — Supporting File [file GEPI-50-0-s001.docx]

**Applying Bayesian multivariable Mendelian randomization to prioritise candidate causal traits from high-dimensional data: Illustration from estimation of the effect of maternal metabolites on offspring birthweight**

**SUPPLEMENTARY MATERIAL**

Ciarrah-Jane Barry^1,2^, Verena Zuber^3,4^, Deborah A Lawlor^1,2,5^, Maria Carolina Borges^1,2^, Eleanor Sanderson^1,2*^, Chin Yang Shapland^1,2*^

*Joint senior authors

^1^ *MRC Integrative Epidemiology Unit, University of Bristol, Bristol, UK.*

*^2^ Population Health Sciences, Bristol Medical School, University of Bristol, Bristol, UK.*

*^3^ MRC Biostatistics Unit, School of Clinical Medicine, University of Cambridge, Cambridge, UK*

*^4^ Department of Epidemiology and Biostatistics, School of Public Health, Imperial College London, London, UK*

*^5^ NIHR Bristol Biomedical Research Centre, Bristol, UK.*

**Consideration of the runtime of different algorithms for MR-BMA**

When few exposures are considered, the exhaustive implementation is preferred since it gives the complete evaluation of the model space. Supplementary Figure 1 gives a comparison of the runtime between the exhaustive and shotgun stochastic search algorithms for MR-BMA for simulated datasets including different numbers of exposures (see Zuber *et al.* [1] for simulations method). While the exhaustive search is computationally extremely fast for smaller model sizes, an exhaustive evaluation is not feasible for too many exposures, since the number of models increases exponentially with respect to the number of exposures. For this comparison, we did evaluate datasets with up to 20 exposures using exhaustive search and additionally 46 exposures to mirror the applied example for shotgun search. The implementation of shotgun stochastic search requires a large number of iterations to ensure convergence. This is why 100 000 iterations should be explored initially to explore the model space. The system.time() function in R is used evaluate the median total elapsed time in seconds across 3 runs.

**Data description**

We used genetic association data from a publicly available GWAS of metabolic traits in up to 115,078 UK Biobank participants of European ancestry (54% females; age (years): mean = 56, SD: 8) available in OpenGWAS[2-4]. Metabolic traits were measured using targeted high-throughput NMR metabolomics (Nightingale Health Ltd., Helsinki, Finland), which provides simultaneous quantification of 249 metabolic traits (i.e., 165 metabolic traits and 84 derived ratios), encompassing routine lipids, lipoprotein subclass profiling (including lipid composition within 14 subclasses), fatty-acid composition, and various low-molecular-weight metabolites such as amino acids, ketone bodies, and glycolysis metabolites Metabolic traits were standardised and normalised prior to analyses using rank-based inverse normal transformation (INT). The GWAS was conducted using linear mixed model (LMM) association method as implemented in BOLT-LMM (v2.3) adjusting for genotype array, fasting time, and sex as previously described.[5]

**Exposure selection**

For the convenience of the reader, we have included the extract about exposure selection from Barry *et al.* (2022):

**“***4.3. Primary Exclusion Criteria for Metabolic Traits to Go into UVMR Analyses*

The NMR platform used in this study includes many lipids and lipoproteins that are known to be closely (i) numerically and/or (ii) biologically related. As an example of (i), concentrations of cholesterol in different lipoprotein particles sum to the concentration of total cholesterol; therefore, total cholesterol is correlated with cholesterol in specific lipoproteins. As an example of (ii), the three glycolysis metabolites (glucose, lactate, and pyruvate) are metabolised via the same biological pathway (i.e., glycolysis), and, as a result, are correlated. This can result in variance inflation and biased estimates of effects in both observational and MVMR analyses if an attempt were made to mutually adjust for all or even a subset of them at the same time, unless one has extremely large sample sizes [58].

Therefore, we applied three exclusion criteria to the full list of 249 NMR metabolic traits to reduce redundancy in our UVMR analyses and avoid multicollinearity in our MVMR models ([Table S7 and Figure S3](https://www.mdpi.com/2218-1989/12/6/537#app1-metabolites-12-00537)).

First, we excluded those with multiple measures that reflected the same metabolic entity (e.g., same trait expressed as a proportion or concentration) or where multiple measures reflected a composite measure of highly related traits (e.g., ‘total fatty acids’ is a composite/combined measure of saturated, monounsaturated, and polyunsaturated fatty acids). As a result, we excluded 84 derived ratios and three composite fatty-acid measures (e.g., total fatty acids, polyunsaturated fatty acids, and degree of unsaturation).

Second, among lipoprotein-related traits, we selected measures related to circulating lipid composition (i.e., total triglycerides, phospholipids, esterified cholesterol, free cholesterol, phosphatidylcholines, and sphingomyelins), apolipoproteins A1 and B, and total lipids in 14 lipoprotein subclasses (i.e., extremely large, very large, large, medium, small, and very small very low-density lipoprotein (VLDL), IDL, large, medium, and small LDL, very large, large, medium, and small HDL). It has been demonstrated previously that we cannot conditionally predict different lipid measures within these subclasses in the same MVMR model due to the very high correlation between these traits [41]. This makes conducting UVMR on different elements within a subclass of lipoprotein redundant as they are not genetically separable and each UVMR would give equivalent estimated effects. As a consequence, we a priori chose to focus on total lipids in each subclass. This selection resulted in the exclusion of 114 measures, mostly representing lipid composition and particle concentration within lipoproteins/lipoprotein subclasses.

Third, we checked the genetic correlation across the remaining measures ([Figure S4](https://www.mdpi.com/2218-1989/12/6/537#app1-metabolites-12-00537)). If two traits are approximately genetically identical, they cannot be distinguished in UVMR analysis or analysed as two distinct traits in a MVMR analysis, due to multicollinearity. Therefore, if a pair of metabolic traits was found to be highly genetically correlated ( > 0.985), one element of the pair was removed. This resulted in the exclusion of two additional metabolic traits (“total lipids in small LDL” and “total lipids in very large VLDL”).”

**Conditional F-statistics**

The conditional F-statistic is a measure used to test for the presence of weak instrument bias in MVMR analysis. This statistic tests the strength of association between the genetic variants and each exposure, conditional on the other exposures included in the analysis. Akin to the F-statistic in a univariable MR analysis, a conditional F-statistic greater than 10 indicates the instruments are less likely to be substantially biased by weak instruments [6, 7].

**Empirical p-values**

Frequentist analysis typically uses hypothesis testing, i.e., comparing whether the test statistic appears under the null or alternative. This lends itself to the calculation of p-values. However, within a Bayesian framework we perform analysis hypothesis free instead considering; posterior probabilities, credible intervals and as here the data and our knowledge about the data to determine the model. It is possible to calculate empirical p-values to provide an indication of how extreme an estimate is relative to a null expectation via permutation[8]. These p-values are calculated from the observed data and do not rely on a theoretical distribution. They are estimated of the probability of observing the data by chance.”

**Supplementary tables**

Table S1: UKBB NMR Metabolites included in analysis

| **Biomarker name** | **Group** | **Subgroup** | **Included** | **Reason for exclusion** |
| --- | --- | --- | --- | --- |
| 3-Hydroxybutyrate | Ketone bodies |  | Yes | -- |
| Acetate | Ketone bodies |  | Yes | -- |
| Acetoacetate | Ketone bodies |  | Yes | -- |
| Acetone | Ketone bodies |  | Yes | -- |
| Alanine | Amino acids |  | Yes | -- |
| Albumin | Fluid balance |  | Yes | -- |
| Apolipoprotein A1 | Apolipoproteins |  | Yes | -- |
| Apolipoprotein B | Apolipoproteins |  | Yes | -- |
| Average diameter for HDL particles | Lipoprotein particle sizes |  | No | Representative of lipid composition within lipoprotein |
| Average diameter for LDL particles | Lipoprotein particle sizes |  | No | Representative of lipid composition within lipoprotein |
| Average diameter for VLDL particles | Lipoprotein particle sizes |  | No | Representative of lipid composition within lipoprotein |
| Cholesterol in chylomicrons and extremely large VLDL | Lipoprotein subclasses | Chylomicrons and extremely large VLDL (particle diameters from 75 nm upwards) | No | Representative of lipid composition within lipoprotein subclass |
| Cholesterol in IDL | Lipoprotein subclasses | IDL (average diameter 28.6 nm) | No | Representative of lipid composition within lipoprotein subclass |
| Cholesterol in large HDL | Lipoprotein subclasses | Large HDL (average diameter 12.1 nm) | No | Representative of lipid composition within lipoprotein subclass |
| Cholesterol in large LDL | Lipoprotein subclasses | Large LDL (average diameter 25.5 nm) | No | Representative of lipid composition within lipoprotein subclass |
| Cholesterol in large VLDL | Lipoprotein subclasses | Large VLDL (average diameter 53.6 nm) | No | Representative of lipid composition within lipoprotein subclass |
| Cholesterol in medium HDL | Lipoprotein subclasses | Medium HDL (average diameter 10.9 nm) | No | Representative of lipid composition within lipoprotein subclass |
| Cholesterol in medium LDL | Lipoprotein subclasses | Medium LDL (average diameter 23 nm) | No | Representative of lipid composition within lipoprotein subclass |
| Cholesterol in medium VLDL | Lipoprotein subclasses | Medium VLDL (average diameter 44.5 nm) | No | Representative of lipid composition within lipoprotein subclass |
| Cholesterol in small HDL | Lipoprotein subclasses | Small HDL (average diameter 8.7 nm) | No | Representative of lipid composition within lipoprotein subclass |
| Cholesterol in small LDL | Lipoprotein subclasses | Small LDL (average diameter 18.7 nm) | No | Representative of lipid composition within lipoprotein subclass |
| Cholesterol in small VLDL | Lipoprotein subclasses | Small VLDL (average diameter 36.8 nm) | No | Representative of lipid composition within lipoprotein subclass |
| Cholesterol in very large HDL | Lipoprotein subclasses | Very large HDL (average diameter 14.3 nm) | No | Representative of lipid composition within lipoprotein subclass |
| Cholesterol in very large VLDL | Lipoprotein subclasses | Very large VLDL (average diameter 64 nm) | No | Representative of lipid composition within lipoprotein subclass |
| Cholesterol in very small VLDL | Lipoprotein subclasses | Very small VLDL (average diameter 31.3 nm) | No | Representative of lipid composition within lipoprotein subclass |
| Cholesterol to total lipids ratio in chylomicrons and extremely large VLDL | Relative lipoprotein lipid concentrations | Chylomicrons and extremely large VLDL ratios | No | Derived measure |
| Cholesterol to total lipids ratio in IDL | Relative lipoprotein lipid concentrations | IDL ratios | No | Derived measure |
| Cholesterol to total lipids ratio in large HDL | Relative lipoprotein lipid concentrations | Large HDL ratios | No | Derived measure |
| Cholesterol to total lipids ratio in large LDL | Relative lipoprotein lipid concentrations | Large LDL ratios | No | Derived measure |
| Cholesterol to total lipids ratio in large VLDL | Relative lipoprotein lipid concentrations | Large VLDL ratios | No | Derived measure |
| Cholesterol to total lipids ratio in medium HDL | Relative lipoprotein lipid concentrations | Medium HDL ratios | No | Derived measure |
| Cholesterol to total lipids ratio in medium LDL | Relative lipoprotein lipid concentrations | Medium LDL ratios | No | Derived measure |
| Cholesterol to total lipids ratio in medium VLDL | Relative lipoprotein lipid concentrations | Medium VLDL ratios | No | Derived measure |
| Cholesterol to total lipids ratio in small HDL | Relative lipoprotein lipid concentrations | Small HDL ratios | No | Derived measure |
| Cholesterol to total lipids ratio in small LDL | Relative lipoprotein lipid concentrations | Small LDL ratios | No | Derived measure |
| Cholesterol to total lipids ratio in small VLDL | Relative lipoprotein lipid concentrations | Small VLDL ratios | No | Derived measure |
| Cholesterol to total lipids ratio in very large HDL | Relative lipoprotein lipid concentrations | Very large HDL ratios | No | Derived measure |
| Cholesterol to total lipids ratio in very large VLDL | Relative lipoprotein lipid concentrations | Very large VLDL ratios | No | Derived measure |
| Cholesterol to total lipids ratio in very small VLDL | Relative lipoprotein lipid concentrations | Very small VLDL ratios | No | Derived measure |
| Cholesteryl esters in chylomicrons and extremely large VLDL | Lipoprotein subclasses | Chylomicrons and extremely large VLDL (particle diameters from 75 nm upwards) | No | Representative of lipid composition within lipoprotein subclass |
| Cholesteryl esters in HDL | Cholesteryl esters |  | No | Representative of lipid composition within lipoprotein |
| Cholesteryl esters in IDL | Lipoprotein subclasses | IDL (average diameter 28.6 nm) | No | Representative of lipid composition within lipoprotein subclass |
| Cholesteryl esters in large HDL | Lipoprotein subclasses | Large HDL (average diameter 12.1 nm) | No | Representative of lipid composition within lipoprotein subclass |
| Cholesteryl esters in large LDL | Lipoprotein subclasses | Large LDL (average diameter 25.5 nm) | No | Representative of lipid composition within lipoprotein subclass |
| Cholesteryl esters in large VLDL | Lipoprotein subclasses | Large VLDL (average diameter 53.6 nm) | No | Representative of lipid composition within lipoprotein subclass |
| Cholesteryl esters in LDL | Cholesteryl esters |  | No | Representative of lipid composition within lipoprotein |
| Cholesteryl esters in medium HDL | Lipoprotein subclasses | Medium HDL (average diameter 10.9 nm) | No | Representative of lipid composition within lipoprotein subclass |
| Cholesteryl esters in medium LDL | Lipoprotein subclasses | Medium LDL (average diameter 23 nm) | No | Representative of lipid composition within lipoprotein subclass |
| Cholesteryl esters in medium VLDL | Lipoprotein subclasses | Medium VLDL (average diameter 44.5 nm) | No | Representative of lipid composition within lipoprotein subclass |
| Cholesteryl esters in small HDL | Lipoprotein subclasses | Small HDL (average diameter 8.7 nm) | No | Representative of lipid composition within lipoprotein subclass |
| Cholesteryl esters in small LDL | Lipoprotein subclasses | Small LDL (average diameter 18.7 nm) | No | Representative of lipid composition within lipoprotein subclass |
| Cholesteryl esters in small VLDL | Lipoprotein subclasses | Small VLDL (average diameter 36.8 nm) | No | Representative of lipid composition within lipoprotein subclass |
| Cholesteryl esters in very large HDL | Lipoprotein subclasses | Very large HDL (average diameter 14.3 nm) | No | Representative of lipid composition within lipoprotein subclass |
| Cholesteryl esters in very large VLDL | Lipoprotein subclasses | Very large VLDL (average diameter 64 nm) | No | Representative of lipid composition within lipoprotein subclass |
| Cholesteryl esters in very small VLDL | Lipoprotein subclasses | Very small VLDL (average diameter 31.3 nm) | No | Representative of lipid composition within lipoprotein subclass |
| Cholesteryl esters in VLDL | Cholesteryl esters |  | No | Representative of lipid composition within lipoprotein |
| Cholesteryl esters to total lipids ratio in chylomicrons and extremely large VLDL | Relative lipoprotein lipid concentrations | Chylomicrons and extremely large VLDL ratios | No | Derived measure |
| Cholesteryl esters to total lipids ratio in IDL | Relative lipoprotein lipid concentrations | IDL ratios | No | Derived measure |
| Cholesteryl esters to total lipids ratio in large HDL | Relative lipoprotein lipid concentrations | Large HDL ratios | No | Derived measure |
| Cholesteryl esters to total lipids ratio in large LDL | Relative lipoprotein lipid concentrations | Large LDL ratios | No | Derived measure |
| Cholesteryl esters to total lipids ratio in large VLDL | Relative lipoprotein lipid concentrations | Large VLDL ratios | No | Derived measure |
| Cholesteryl esters to total lipids ratio in medium HDL | Relative lipoprotein lipid concentrations | Medium HDL ratios | No | Derived measure |
| Cholesteryl esters to total lipids ratio in medium LDL | Relative lipoprotein lipid concentrations | Medium LDL ratios | No | Derived measure |
| Cholesteryl esters to total lipids ratio in medium VLDL | Relative lipoprotein lipid concentrations | Medium VLDL ratios | No | Derived measure |
| Cholesteryl esters to total lipids ratio in small HDL | Relative lipoprotein lipid concentrations | Small HDL ratios | No | Derived measure |
| Cholesteryl esters to total lipids ratio in small LDL | Relative lipoprotein lipid concentrations | Small LDL ratios | No | Derived measure |
| Cholesteryl esters to total lipids ratio in small VLDL | Relative lipoprotein lipid concentrations | Small VLDL ratios | No | Derived measure |
| Cholesteryl esters to total lipids ratio in very large HDL | Relative lipoprotein lipid concentrations | Very large HDL ratios | No | Derived measure |
| Cholesteryl esters to total lipids ratio in very large VLDL | Relative lipoprotein lipid concentrations | Very large VLDL ratios | No | Derived measure |
| Cholesteryl esters to total lipids ratio in very small VLDL | Relative lipoprotein lipid concentrations | Very small VLDL ratios | No | Derived measure |
| Citrate | Glycolysis related metabolites |  | Yes | -- |
| Clinical LDL cholesterol | Cholesterol |  | No | Representative of lipid composition within lipoprotein |
| Concentration of chylomicrons and extremely large VLDL particles | Lipoprotein subclasses | Chylomicrons and extremely large VLDL (particle diameters from 75 nm upwards) | No | Representative of lipid composition within lipoprotein subclass |
| Concentration of HDL particles | Lipoprotein particle concentrations |  | No | Representative of lipid concentration within lipoprotein subclass |
| Concentration of IDL particles | Lipoprotein subclasses | IDL (average diameter 28.6 nm) | No | Representative of lipid concentration within lipoprotein subclass |
| Concentration of large HDL particles | Lipoprotein subclasses | Large HDL (average diameter 12.1 nm) | No | Representative of lipid concentration within lipoprotein subclass |
| Concentration of large LDL particles | Lipoprotein subclasses | Large LDL (average diameter 25.5 nm) | No | Representative of lipid composition within lipoprotein subclass |
| Concentration of large VLDL particles | Lipoprotein subclasses | Large VLDL (average diameter 53.6 nm) | No | Representative of lipid composition within lipoprotein subclass |
| Concentration of LDL particles | Lipoprotein particle concentrations |  | No | Representative of lipid concentration within lipoprotein subclass |
| Concentration of medium HDL particles | Lipoprotein subclasses | Medium HDL (average diameter 10.9 nm) | No | Representative of lipid composition within lipoprotein subclass |
| Concentration of medium LDL particles | Lipoprotein subclasses | Medium LDL (average diameter 23 nm) | No | Representative of lipid composition within lipoprotein subclass |
| Concentration of medium VLDL particles | Lipoprotein subclasses | Medium VLDL (average diameter 44.5 nm) | No | Representative of lipid composition within lipoprotein subclass |
| Concentration of small HDL particles | Lipoprotein subclasses | Small HDL (average diameter 8.7 nm) | No | Representative of lipid composition within lipoprotein subclass |
| Concentration of small LDL particles | Lipoprotein subclasses | Small LDL (average diameter 18.7 nm) | No | Representative of lipid composition within lipoprotein subclass |
| Concentration of small VLDL particles | Lipoprotein subclasses | Small VLDL (average diameter 36.8 nm) | No | Representative of lipid composition within lipoprotein subclass |
| Concentration of very large HDL particles | Lipoprotein subclasses | Very large HDL (average diameter 14.3 nm) | No | Representative of lipid composition within lipoprotein subclass |
| Concentration of very large VLDL particles | Lipoprotein subclasses | Very large VLDL (average diameter 64 nm) | No | Representative of lipid composition within lipoprotein subclass |
| Concentration of very small VLDL particles | Lipoprotein subclasses | Very small VLDL (average diameter 31.3 nm) | No | Representative of lipid composition within lipoprotein subclass |
| Concentration of VLDL particles | Lipoprotein particle concentrations |  | No | Representative of lipid concentration within lipoprotein subclass |
| Creatinine | Fluid balance |  | Yes | -- |
| Degree of unsaturation | Fatty acids |  | No | Composite fatty acid measure |
| Docosahexaenoic acid | Fatty acids |  | Yes | -- |
| Free cholesterol in chylomicrons and extremely large VLDL | Lipoprotein subclasses | Chylomicrons and extremely large VLDL (particle diameters from 75 nm upwards) | No | Representative of lipid composition within lipoprotein subclass |
| Free cholesterol in HDL | Free cholesterol |  | No | Representative of lipid composition within lipoprotein |
| Free cholesterol in IDL | Lipoprotein subclasses | IDL (average diameter 28.6 nm) | No | Representative of lipid composition within lipoprotein subclass |
| Free cholesterol in large HDL | Lipoprotein subclasses | Large HDL (average diameter 12.1 nm) | No | Representative of lipid composition within lipoprotein subclass |
| Free cholesterol in large LDL | Lipoprotein subclasses | Large LDL (average diameter 25.5 nm) | No | Representative of lipid composition within lipoprotein subclass |
| Free cholesterol in large VLDL | Lipoprotein subclasses | Large VLDL (average diameter 53.6 nm) | No | Representative of lipid composition within lipoprotein subclass |
| Free cholesterol in LDL | Free cholesterol |  | No | Representative of lipid composition within lipoprotein |
| Free cholesterol in medium HDL | Lipoprotein subclasses | Medium HDL (average diameter 10.9 nm) | No | Representative of lipid composition within lipoprotein subclass |
| Free cholesterol in medium LDL | Lipoprotein subclasses | Medium LDL (average diameter 23 nm) | No | Representative of lipid composition within lipoprotein subclass |
| Free cholesterol in medium VLDL | Lipoprotein subclasses | Medium VLDL (average diameter 44.5 nm) | No | Representative of lipid composition within lipoprotein subclass |
| Free cholesterol in small HDL | Lipoprotein subclasses | Small HDL (average diameter 8.7 nm) | No | Representative of lipid composition within lipoprotein subclass |
| Free cholesterol in small LDL | Lipoprotein subclasses | Small LDL (average diameter 18.7 nm) | No | Representative of lipid composition within lipoprotein subclass |
| Free cholesterol in small VLDL | Lipoprotein subclasses | Small VLDL (average diameter 36.8 nm) | No | Representative of lipid composition within lipoprotein subclass |
| Free cholesterol in very large HDL | Lipoprotein subclasses | Very large HDL (average diameter 14.3 nm) | No | Representative of lipid composition within lipoprotein subclass |
| Free cholesterol in very large VLDL | Lipoprotein subclasses | Very large VLDL (average diameter 64 nm) | No | Representative of lipid composition within lipoprotein subclass |
| Free cholesterol in very small VLDL | Lipoprotein subclasses | Very small VLDL (average diameter 31.3 nm) | No | Representative of lipid composition within lipoprotein subclass |
| Free cholesterol in VLDL | Free cholesterol |  | No | Representative of lipid composition within lipoprotein |
| Free cholesterol to total lipids ratio in chylomicrons and extremely large VLDL | Relative lipoprotein lipid concentrations | Chylomicrons and extremely large VLDL ratios | No | Derived measure |
| Free cholesterol to total lipids ratio in IDL | Relative lipoprotein lipid concentrations | IDL ratios | No | Derived measure |
| Free cholesterol to total lipids ratio in large HDL | Relative lipoprotein lipid concentrations | Large HDL ratios | No | Derived measure |
| Free cholesterol to total lipids ratio in large LDL | Relative lipoprotein lipid concentrations | Large LDL ratios | No | Derived measure |
| Free cholesterol to total lipids ratio in large VLDL | Relative lipoprotein lipid concentrations | Large VLDL ratios | No | Derived measure |
| Free cholesterol to total lipids ratio in medium HDL | Relative lipoprotein lipid concentrations | Medium HDL ratios | No | Derived measure |
| Free cholesterol to total lipids ratio in medium LDL | Relative lipoprotein lipid concentrations | Medium LDL ratios | No | Derived measure |
| Free cholesterol to total lipids ratio in medium VLDL | Relative lipoprotein lipid concentrations | Medium VLDL ratios | No | Derived measure |
| Free cholesterol to total lipids ratio in small HDL | Relative lipoprotein lipid concentrations | Small HDL ratios | No | Derived measure |
| Free cholesterol to total lipids ratio in small LDL | Relative lipoprotein lipid concentrations | Small LDL ratios | No | Derived measure |
| Free cholesterol to total lipids ratio in small VLDL | Relative lipoprotein lipid concentrations | Small VLDL ratios | No | Derived measure |
| Free cholesterol to total lipids ratio in very large HDL | Relative lipoprotein lipid concentrations | Very large HDL ratios | No | Derived measure |
| Free cholesterol to total lipids ratio in very large VLDL | Relative lipoprotein lipid concentrations | Very large VLDL ratios | No | Derived measure |
| Free cholesterol to total lipids ratio in very small VLDL | Relative lipoprotein lipid concentrations | Very small VLDL ratios | No | Derived measure |
| Glucose | Glycolysis related metabolites |  | Yes | -- |
| Glutamine | Amino acids |  | Yes | -- |
| Glycine | Amino acids |  | Yes | -- |
| Glycoprotein acetyls | Inflammation |  | Yes | -- |
| HDL cholesterol | Cholesterol |  | No | Representative of lipid composition within lipoprotein |
| Histidine | Amino acids |  | Yes | -- |
| Isoleucine | Amino acids | Branched-chain amino acids | Yes | -- |
| Lactate | Glycolysis related metabolites |  | Yes | -- |
| LDL cholesterol | Cholesterol |  | No | Representative of lipid composition within lipoprotein |
| Leucine | Amino acids | Branched-chain amino acids | Yes | -- |
| Linoleic acid | Fatty acids |  | Yes | -- |
| Monounsaturated fatty acids | Fatty acids |  | Yes | -- |
| Omega-3 fatty acids | Fatty acids |  | Yes | -- |
| Omega-6 fatty acids | Fatty acids |  | Yes | -- |
| Phenylalanine | Amino acids | Aromatic amino acids | Yes | -- |
| Phosphatidylcholines | Other lipids |  | Yes | -- |
| Phosphoglycerides | Other lipids |  | No | Representative of lipid composition within lipoprotein |
| Phospholipids in chylomicrons and extremely large VLDL | Lipoprotein subclasses | Chylomicrons and extremely large VLDL (particle diameters from 75 nm upwards) | No | Representative of lipid composition within lipoprotein subclass |
| Phospholipids in HDL | Phospholipids |  | No | Representative of lipid composition within lipoprotein subclass |
| Phospholipids in IDL | Lipoprotein subclasses | IDL (average diameter 28.6 nm) | No | Representative of lipid composition within lipoprotein subclasses |
| Phospholipids in large HDL | Lipoprotein subclasses | Large HDL (average diameter 12.1 nm) | No | Representative of lipid composition within lipoprotein subclasses |
| Phospholipids in large LDL | Lipoprotein subclasses | Large LDL (average diameter 25.5 nm) | No | Representative of lipid composition within lipoprotein subclasses |
| Phospholipids in large VLDL | Lipoprotein subclasses | Large VLDL (average diameter 53.6 nm) | No | Representative of lipid composition within lipoprotein subclasses |
| Phospholipids in LDL | Phospholipids |  | No | Representative of lipid composition within lipoprotein subclass |
| Phospholipids in medium HDL | Lipoprotein subclasses | Medium HDL (average diameter 10.9 nm) | No | Representative of lipid composition within lipoprotein subclasses |
| Phospholipids in medium LDL | Lipoprotein subclasses | Medium LDL (average diameter 23 nm) | No | Representative of lipid composition within lipoprotein subclasses |
| Phospholipids in medium VLDL | Lipoprotein subclasses | Medium VLDL (average diameter 44.5 nm) | No | Representative of lipid composition within lipoprotein subclasses |
| Phospholipids in small HDL | Lipoprotein subclasses | Small HDL (average diameter 8.7 nm) | No | Representative of lipid composition within lipoprotein subclasses |
| Phospholipids in small LDL | Lipoprotein subclasses | Small LDL (average diameter 18.7 nm) | No | Representative of lipid composition within lipoprotein subclasses |
| Phospholipids in small VLDL | Lipoprotein subclasses | Small VLDL (average diameter 36.8 nm) | No | Representative of lipid composition within lipoprotein subclasses |
| Phospholipids in very large HDL | Lipoprotein subclasses | Very large HDL (average diameter 14.3 nm) | No | Representative of lipid composition within lipoprotein subclasses |
| Phospholipids in very large VLDL | Lipoprotein subclasses | Very large VLDL (average diameter 64 nm) | No | Representative of lipid composition within lipoprotein subclasses |
| Phospholipids in very small VLDL | Lipoprotein subclasses | Very small VLDL (average diameter 31.3 nm) | No | Representative of lipid composition within lipoprotein subclasses |
| Phospholipids in VLDL | Phospholipids |  | No | Representative of lipid composition within lipoprotein subclass |
| Phospholipids to total lipids ratio in chylomicrons and extremely large VLDL | Relative lipoprotein lipid concentrations | Chylomicrons and extremely large VLDL ratios | No | Derived measure |
| Phospholipids to total lipids ratio in IDL | Relative lipoprotein lipid concentrations | IDL ratios | No | Derived measure |
| Phospholipids to total lipids ratio in large HDL | Relative lipoprotein lipid concentrations | Large HDL ratios | No | Derived measure |
| Phospholipids to total lipids ratio in large LDL | Relative lipoprotein lipid concentrations | Large LDL ratios | No | Derived measure |
| Phospholipids to total lipids ratio in large VLDL | Relative lipoprotein lipid concentrations | Large VLDL ratios | No | Derived measure |
| Phospholipids to total lipids ratio in medium HDL | Relative lipoprotein lipid concentrations | Medium HDL ratios | No | Derived measure |
| Phospholipids to total lipids ratio in medium LDL | Relative lipoprotein lipid concentrations | Medium LDL ratios | No | Derived measure |
| Phospholipids to total lipids ratio in medium VLDL | Relative lipoprotein lipid concentrations | Medium VLDL ratios | No | Derived measure |
| Phospholipids to total lipids ratio in small HDL | Relative lipoprotein lipid concentrations | Small HDL ratios | No | Derived measure |
| Phospholipids to total lipids ratio in small LDL | Relative lipoprotein lipid concentrations | Small LDL ratios | No | Derived measure |
| Phospholipids to total lipids ratio in small VLDL | Relative lipoprotein lipid concentrations | Small VLDL ratios | No | Derived measure |
| Phospholipids to total lipids ratio in very large HDL | Relative lipoprotein lipid concentrations | Very large HDL ratios | No | Derived measure |
| Phospholipids to total lipids ratio in very large VLDL | Relative lipoprotein lipid concentrations | Very large VLDL ratios | No | Derived measure |
| Phospholipids to total lipids ratio in very small VLDL | Relative lipoprotein lipid concentrations | Very small VLDL ratios | No | Derived measure |
| Polyunsaturated fatty acids | Fatty acids |  | No | Composite fatty acid measure |
| Pyruvate | Glycolysis related metabolites |  | Yes | -- |
| Ratio of apolipoprotein B to apolipoprotein A1 | Apolipoproteins |  | No | Derived measure |
| Ratio of docosahexaenoic acid to total fatty acids | Fatty acids | Fatty acid ratios | No | Derived measure |
| Ratio of linoleic acid to total fatty acids | Fatty acids | Fatty acid ratios | No | Derived measure |
| Ratio of monounsaturated fatty acids to total fatty acids | Fatty acids | Fatty acid ratios | No | Derived measure |
| Ratio of omega-3 fatty acids to total fatty acids | Fatty acids | Fatty acid ratios | No | Derived measure |
| Ratio of omega-6 fatty acids to omega-3 fatty acids | Fatty acids | Fatty acid ratios | No | Derived measure |
| Ratio of omega-6 fatty acids to total fatty acids | Fatty acids | Fatty acid ratios | No | Derived measure |
| Ratio of polyunsaturated fatty acids to monounsaturated fatty acids | Fatty acids | Fatty acid ratios | No | Derived measure |
| Ratio of polyunsaturated fatty acids to total fatty acids | Fatty acids | Fatty acid ratios | No | Derived measure |
| Ratio of saturated fatty acids to total fatty acids | Fatty acids | Fatty acid ratios | No | Derived measure |
| Ratio of triglycerides to phosphoglycerides | Other lipids |  | No | Derived measure |
| Remnant cholesterol (non-HDL, non-LDL -cholesterol) | Cholesterol |  | No | Derived measure |
| Saturated fatty acids | Fatty acids |  | Yes | ---- |
| Sphingomyelins | Other lipids |  | Yes |  |
| Total cholesterol | Cholesterol |  | No |  |
| Total cholesterol minus HDL-C | Cholesterol |  | No | Derived measure |
| Total cholines | Other lipids |  | No |  |
| Total concentration of branched-chain amino acids (leucine + isoleucine + valine) | Amino acids | Branched-chain amino acids | No | Derived measure |
| Total concentration of lipoprotein particles | Lipoprotein particle concentrations |  | No |  |
| Total esterified cholesterol | Cholesteryl esters |  | Yes | -- |
| Total fatty acids | Fatty acids |  | No | Composite fatty acid measure |
| Total free cholesterol | Free cholesterol |  | Yes | -- |
| Total lipids in chylomicrons and extremely large VLDL | Lipoprotein subclasses | Chylomicrons and extremely large VLDL (particle diameters from 75 nm upwards) | Yes | -- |
| Total lipids in HDL | Total lipids |  | No |  |
| Total lipids in IDL | Lipoprotein subclasses | IDL (average diameter 28.6 nm) | Yes | -- |
| Total lipids in large HDL | Lipoprotein subclasses | Large HDL (average diameter 12.1 nm) | Yes | -- |
| Total lipids in large LDL | Lipoprotein subclasses | Large LDL (average diameter 25.5 nm) | Yes | -- |
| Total lipids in large VLDL | Lipoprotein subclasses | Large VLDL (average diameter 53.6 nm) | Yes | -- |
| Total lipids in LDL | Total lipids |  | No |  |
| Total lipids in lipoprotein particles | Total lipids |  | No |  |
| Total lipids in medium HDL | Lipoprotein subclasses | Medium HDL (average diameter 10.9 nm) | Yes | -- |
| Total lipids in medium LDL | Lipoprotein subclasses | Medium LDL (average diameter 23 nm) | Yes | -- |
| Total lipids in medium VLDL | Lipoprotein subclasses | Medium VLDL (average diameter 44.5 nm) | Yes | -- |
| Total lipids in small HDL | Lipoprotein subclasses | Small HDL (average diameter 8.7 nm) | Yes | -- |
| Total lipids in small LDL | Lipoprotein subclasses | Small LDL (average diameter 18.7 nm) | No | High correlation |
| Total lipids in small VLDL | Lipoprotein subclasses | Small VLDL (average diameter 36.8 nm) | Yes | -- |
| Total lipids in very large HDL | Lipoprotein subclasses | Very large HDL (average diameter 14.3 nm) | Yes | -- |
| Total lipids in very large VLDL | Lipoprotein subclasses | Very large VLDL (average diameter 64 nm) | No | High correlation |
| Total lipids in very small VLDL | Lipoprotein subclasses | Very small VLDL (average diameter 31.3 nm) | Yes | -- |
| Total lipids in VLDL | Total lipids |  | No |  |
| Total phospholipids in lipoprotein particles | Phospholipids |  | Yes | -- |
| Total triglycerides | Triglycerides |  | Yes | -- |
| Triglycerides in chylomicrons and extremely large VLDL | Lipoprotein subclasses | Chylomicrons and extremely large VLDL (particle diameters from 75 nm upwards) | No |  |
| Triglycerides in HDL | Triglycerides |  | No |  |
| Triglycerides in IDL | Lipoprotein subclasses | IDL (average diameter 28.6 nm) | No |  |
| Triglycerides in large HDL | Lipoprotein subclasses | Large HDL (average diameter 12.1 nm) | No |  |
| Triglycerides in large LDL | Lipoprotein subclasses | Large LDL (average diameter 25.5 nm) | No |  |
| Triglycerides in large VLDL | Lipoprotein subclasses | Large VLDL (average diameter 53.6 nm) | No |  |
| Triglycerides in LDL | Triglycerides |  | No |  |
| Triglycerides in medium HDL | Lipoprotein subclasses | Medium HDL (average diameter 10.9 nm) | No |  |
| Triglycerides in medium LDL | Lipoprotein subclasses | Medium LDL (average diameter 23 nm) | No |  |
| Triglycerides in medium VLDL | Lipoprotein subclasses | Medium VLDL (average diameter 44.5 nm) | No |  |
| Triglycerides in small HDL | Lipoprotein subclasses | Small HDL (average diameter 8.7 nm) | No |  |
| Triglycerides in small LDL | Lipoprotein subclasses | Small LDL (average diameter 18.7 nm) | No |  |
| Triglycerides in small VLDL | Lipoprotein subclasses | Small VLDL (average diameter 36.8 nm) | No |  |
| Triglycerides in very large HDL | Lipoprotein subclasses | Very large HDL (average diameter 14.3 nm) | No |  |
| Triglycerides in very large VLDL | Lipoprotein subclasses | Very large VLDL (average diameter 64 nm) | No |  |
| Triglycerides in very small VLDL | Lipoprotein subclasses | Very small VLDL (average diameter 31.3 nm) | No |  |
| Triglycerides in VLDL | Triglycerides |  | No |  |
| Triglycerides to total lipids ratio in chylomicrons and extremely large VLDL | Relative lipoprotein lipid concentrations | Chylomicrons and extremely large VLDL ratios | No | Derived measure |
| Triglycerides to total lipids ratio in IDL | Relative lipoprotein lipid concentrations | IDL ratios | No | Derived measure |
| Triglycerides to total lipids ratio in large HDL | Relative lipoprotein lipid concentrations | Large HDL ratios | No | Derived measure |
| Triglycerides to total lipids ratio in large LDL | Relative lipoprotein lipid concentrations | Large LDL ratios | No | Derived measure |
| Triglycerides to total lipids ratio in large VLDL | Relative lipoprotein lipid concentrations | Large VLDL ratios | No | Derived measure |
| Triglycerides to total lipids ratio in medium HDL | Relative lipoprotein lipid concentrations | Medium HDL ratios | No | Derived measure |
| Triglycerides to total lipids ratio in medium LDL | Relative lipoprotein lipid concentrations | Medium LDL ratios | No | Derived measure |
| Triglycerides to total lipids ratio in medium VLDL | Relative lipoprotein lipid concentrations | Medium VLDL ratios | No | Derived measure |
| Triglycerides to total lipids ratio in small HDL | Relative lipoprotein lipid concentrations | Small HDL ratios | No | Derived measure |
| Triglycerides to total lipids ratio in small LDL | Relative lipoprotein lipid concentrations | Small LDL ratios | No | Derived measure |
| Triglycerides to total lipids ratio in small VLDL | Relative lipoprotein lipid concentrations | Small VLDL ratios | No | Derived measure |
| Triglycerides to total lipids ratio in very large HDL | Relative lipoprotein lipid concentrations | Very large HDL ratios | No | Derived measure |
| Triglycerides to total lipids ratio in very large VLDL | Relative lipoprotein lipid concentrations | Very large VLDL ratios | No | Derived measure |
| Triglycerides to total lipids ratio in very small VLDL | Relative lipoprotein lipid concentrations | Very small VLDL ratios | No | Derived measure |
| Tyrosine | Amino acids | Aromatic amino acids | Yes | -- |
| Valine | Amino acids | Branched-chain amino acids | Yes | -- |
| VLDL cholesterol | Cholesterol |  | No | Representative of lipid composition within lipoprotein subclass |

*Table S2: The ranked of sets of univariate exposures for birthweight according to their PP for the top 50 models. Model specific causal estimates to be interpreted with caution due to likely attenuation towards the null.*

| **Model** | **Posterior probability (PP)** | **Model specific causal estimates** |
| --- | --- | --- |
| Glutamine, Glucose | 0.37666 | 0.0576, 0.23651 |
| Glucose | 0.3109 | 0.2441 |
| Alanine, Glutamine, Glucose | 0.05121 | 0.0665, 0.05779, 0.22074 |
| Alanine, Glucose | 0.03782 | 0.06611, 0.22845 |
| Glutamine, Glucose, Lactate | 0.0213 | 0.06017, 0.25436, 0.07595 |
| Glucose, Lactate | 0.00998 | 0.25997, 0.06628 |
| Glucose, Total lipids in small HDL | 0.00732 | 0.25384, -0.03464 |
| Acetate, Glutamine, Glucose | 0.00729 | -0.05653, 0.06087, 0.24267 |
| Acetoacetate, Glucose | 0.0048 | -0.05016, 0.24888 |
| Acetoacetate, Glutamine, Glucose | 0.00419 | -0.04014, 0.05613, 0.24052 |
| Glutamine, Glucose, Total lipids in small HDL | 0.004 | 0.05399, 0.24468, -0.02736 |
| Glucose, Total lipids in medium HDL | 0.00399 | 0.24736, -0.02597 |
| Glutamine, Glucose, Total lipids in medium HDL | 0.00392 | 0.05649, 0.23968, -0.0241 |
| Acetate, Glucose | 0.00323 | -0.03829, 0.24857 |
| Glucose, Glycine | 0.00309 | 0.24433, 0.01843 |
| Alanine, Glucose, Total lipids in small HDL | 0.00285 | 0.07734, 0.23796, -0.04327 |
| Apolipoprotein A1, Glutamine, Glucose | 0.00284 | -0.02126, 0.0569, 0.23911 |
| Glutamine, Glucose, Glycine | 0.00278 | 0.05609, 0.23691, 0.01672 |
| Acetone, Glutamine, Glucose | 0.00276 | -0.02477, 0.05826, 0.23547 |
| Apolipoprotein A1, Glucose | 0.00266 | -0.02258, 0.24677 |
| Albumin, Glucose | 0.00245 | 0.02785, 0.23981 |
| Glucose, Histidine | 0.0024 | 0.2413, 0.02842 |
| Glutamine, Glucose, Pyruvate | 0.00231 | 0.05675, 0.23797, -0.02171 |
| Glucose, Pyruvate | 0.0023 | 0.2458, -0.02725 |
| Albumin, Glutamine, Glucose | 0.00226 | 0.02179, 0.05639, 0.23331 |
| Glutamine, Glucose, Leucine | 0.00225 | 0.05816, 0.2332, 0.02014 |
| Creatinine, Glutamine, Glucose | 0.00214 | -0.02028, 0.0578, 0.23933 |
| Citrate, Glutamine, Glucose | 0.00207 | -0.01985, 0.05927, 0.23806 |
| Acetone, Glucose | 0.002 | -0.01824, 0.24341 |
| Glutamine, Glucose, Isoleucine | 0.00197 | 0.05773, 0.2358, 0.00369 |
| Glutamine, Glucose, Phenylalanine | 0.00184 | 0.0579, 0.23715, -0.00777 |
| 3-Hydroxybutyrate, Glutamine, Glucose | 0.00183 | -0.00687, 0.05745, 0.23637 |
| Glutamine, Glucose, Histidine | 0.00183 | 0.05656, 0.23586, 0.00798 |
| Creatinine, Glucose | 0.00168 | -0.01907, 0.24678 |
| Glucose, Leucine | 0.00166 | 0.24159, 0.01563 |
| Glucose, Isoleucine | 0.00162 | 0.24479, -0.00365 |
| 3-Hydroxybutyrate, Glucose | 0.00157 | -0.01058, 0.24387 |
| Glutamine, Glucose, Valine | 0.00156 | 0.05772, 0.23588, 0.00239 |
| Glutamine, Glucose, Tyrosine | 0.00149 | 0.0573, 0.23453, 0.01237 |
| Docosahexaenoic acid, Glutamine, Glucose | 0.00147 | -0.01425, 0.05695, 0.23972 |
| Glucose, Phenylalanine | 0.00146 | 0.24421, -0.00124 |
| Alanine, Glutamine, Glucose, Total lipids in small HDL | 0.0014 | 0.0758, 0.05307, 0.22927, -0.03594 |
| Docosahexaenoic acid, Glucose | 0.00139 | -0.01605, 0.24763 |
| Glutamine, Glucose, GlycinecA | 0.00139 | 0.0596, 0.23283, 0.013 |
| Apolipoprotein B, Glutamine, Glucose | 0.00133 | 0.01291, 0.05829, 0.23402 |
| Glucose, Tyrosine | 0.00131 | 0.24177, 0.0143 |
| Glucose, Valine | 0.0013 | 0.24526, -0.00448 |
| Citrate, Glucose | 0.00125 | -0.01393, 0.24535 |
| Glutamine, Glucose, Total lipids in large VLDL | 0.0012 | 0.05918, 0.23221, 0.01134 |
| Glutamine, Glucose, Total lipids in medium VLDL | 0.0012 | 0.05884, 0.23271, 0.01125 |

*Table S3: The ranked of sets of exposures for birthweight according to their marginal inclusion probability. Model specific causal estimates to be interpreted with caution due to likely attenuation towards the null. Empirical p-values give an indication of how extreme an estimate is relative to a null expectation via permutation.*

| **Exposure** | **Marginal inclusion probability (MIP)** | **Model averaged causal effect** | **Empirical p-values** |
| --- | --- | --- | --- |
| Glucose | 1 | 0.23915 | <0.001 |
| Glutamine | 0.5511 | 0.03181 | 0.005 |
| Alanine | 0.11699 | 0.00787 | 0.037 |
| Lactate | 0.04095 | 0.00298 | 0.274 |
| Total lipids in small HDL | 0.02096 | -0.00075 | 0.249 |
| Acetate | 0.0141 | -7.00E-04 | 0.930 |
| Total lipids in medium HDL | 0.01254 | -0.00034 | 0.554 |
| Acetoacetate | 0.01205 | -0.00053 | 0.953 |
| Glycine | 0.00873 | 0.00016 | 0.451 |
| Apolipoprotein A1 | 0.0087 | -0.00019 | 0.822 |
| Pyruvate | 0.0071 | -0.00019 | 0.968 |
| Acetone | 0.00664 | -0.00013 | 0.994 |
| Albumin | 0.00653 | 0.00016 | 0.962 |
| Isoleucine | 0.00617 | -0.00012 | 0.995 |
| Leucine | 0.00608 | 0.00017 | 0.991 |
| Histidine | 0.00594 | 1.00E-04 | 0.988 |
| Creatinine | 0.00556 | -0.00011 | 0.983 |
| 3-Hydroxybutyrate | 0.00486 | -4.00E-05 | 0.999 |
| Phenylalanine | 0.00486 | -4.00E-05 | 0.998 |
| Citrate | 0.00485 | -9.00E-05 | 0.966 |
| Valine | 0.0048 | -6.00E-05 | 0.997 |
| Docosahexaenoic acid | 0.00431 | -7.00E-05 | 0.970 |
| Tyrosine | 0.00388 | 5.00E-05 | 0.990 |
| Apolipoprotein B | 0.00342 | 5.00E-05 | 0.986 |
| Phosphatidylcholines | 0.00339 | -4.00E-05 | 0.991 |
| Omega-3 fatty acids | 0.00333 | -4.00E-05 | 0.978 |
| GlycinecA | 0.00319 | 3.00E-05 | 0.996 |
| Total lipids in medium LDL | 0.00298 | 3.00E-05 | 0.996 |
| Total phospholipids in lipoprotein particles | 0.00297 | -3.00E-05 | 0.993 |
| Total lipids in medium VLDL | 0.00292 | 3.00E-05 | 0.994 |
| Total lipids in large VLDL | 0.00287 | 3.00E-05 | 0.995 |
| Saturated fatty acids | 0.00277 | 0 | 0.999 |
| Monounsaturated fatty acids | 0.00268 | 2.00E-05 | 0.998 |
| Total lipids in large LDL | 0.00267 | 2.00E-05 | 0.997 |
| Omega-6 fatty acids | 0.00262 | -1.00E-05 | 0.999 |
| Total free cholesterol | 0.00261 | 1.00E-05 | 0.999 |
| Linoleic acid | 0.0026 | 1.00E-05 | 0.999 |
| Total lipids in IDL | 0.00256 | 2.00E-05 | 0.997 |
| Total lipids in large HDL | 0.00255 | -1.00E-05 | 0.994 |
| Total triglycerides | 0.0025 | 1.00E-05 | 0.998 |
| Total esterified cholesterol | 0.00249 | 0 | 1.000 |
| Total lipids in chylomicrons and extremely large VLDL | 0.00247 | 1.00E-05 | 0.998 |
| Total lipids in small VLDL | 0.0024 | 1.00E-05 | 0.999 |
| Sphingomyelins | 0.00236 | 0 | 1.000 |
| Total lipids in very small VLDL | 0.00226 | 1.00E-05 | 0.998 |
| Total lipids in very large HDL | 0.00224 | 2.00E-05 | 0.998 |

*Table S4a, b: We hold the prior variance constant at default* $(\sigma^{2}=0.5)$ *and vary the prior probability p,* $p=(0.01, 0.05, 0.10, 0.20$*) to check the robustness of results and sensitivity to model selection parameters for the top 10 ranked sets of exposures. The top 10 ranked of sets of exposures for birthweight according to their posterior probability (a) and marginal inclusion probability (b).*

*a)*

| **p=0.01** | | | **p=0.05** | | | **p=0.1** | | | **p=0.2** | | |
| --- | --- | --- | --- | --- | --- | --- | --- | --- | --- | --- | --- |
| **Model** | **Posterior probability (PP)** | **Model specific causal estimates** | **Model** | **Posterior probability (PP)** | **Model specific causal estimates** | **Model** | **Posterior probability (PP)** | **Model specific causal estimates** | **Model** | **Posterior probability (PP)** | **Model specific causal estimates** |
| Glucose | 0.871 | 0.244 | Glucose | 0.532 | 0.244 | Glutamine,Glucose | 0.377 | 0.058, 0.237 | Glutamine, Glucose | 0.318 | 0.058, 0.237 |
| Glutamine, Glucose | 0.096 | 0.058, 0.237 | Glutamine, Glucose | 0.306 | 0.058, 0.237 | Glucose | 0.311 | 0.244 | Glucose | 0.117 | 0.244 |
| Alanine, Glucose | 0.01 | 0.066, 0.228 | Alanine, Glucose | 0.031 | 0.066, 0.228 | Alanine, Glutamine, Glucose | 0.051 | 0.067, 0.058, 0.221 | Alanine, Glutamine, Glucose | 0.097 | 0.067, 0.058, 0.221 |
| Glucose, Lactate | 0.003 | 0.26, 0.066 | Alanine, Glutamine, Glucose | 0.02 | 0.067, 0.058, 0.221 | Alanine, Glucose | 0.038 | 0.066, 0.228 | Glutamine, Glucose, Lactate | 0.041 | 0.06, 0.254, 0.076 |
| Glucose, Total lipids in small HDL | 0.002 | 0.254, -0.035 | Glutamine, Glucose, Lactate | 0.008 | 0.06, 0.254, 0.076 | Glutamine, Glucose, Lactate | 0.021 | 0.06, 0.254, 0.076 | Alanine, Glucose | 0.032 | 0.066, 0.228 |
| Acetoacetate, Glucose | 0.001 | -0.05, 0.249 | Glucose, Lactate | 0.008 | 0.26, 0.066 | Glucose, Lactate | 0.01 | 0.26, 0.066 | Acetate, Glutamine, Glucose | 0.014 | -0.057, 0.061, 0.243 |
| Alanine, Glutamine, Glucose | 0.001 | 0.067, 0.058, 0.221 | Glucose, Total lipids in small HDL | 0.006 | 0.254, -0.035 | Glucose, Total lipids in small HDL | 0.007 | 0.254, -0.035 | Glucose, Lactate | 0.008 | 0.26, 0.066 |
| Glucose, Total lipids in medium HDL | 0.001 | 0.247, -0.026 | Acetoacetate, Glucose | 0.004 | -0.05, 0.249 | Acetate, Glutamine, Glucose | 0.007 | -0.057, 0.061, 0.243 | Acetoacetate, Glutamine, Glucose | 0.008 | -0.04, 0.056, 0.241 |
| Acetate, Glucose | 0.001 | -0.038, 0.249 | Glucose, Total lipids in medium HDL | 0.003 | 0.247, -0.026 | Acetoacetate, Glucose | 0.005 | -0.05, 0.249 | Glutamine, Glucose, Total lipids in small HDL | 0.008 | 0.054, 0.245, -0.027 |
| Glucose, Glycine | 0.001 | 0.244, 0.018 | Acetate, Glutamine, Glucose | 0.003 | -0.057, 0.061, 0.243 | Acetoacetate, Glutamine, Glucose | 0.004 | -0.04, 0.056, 0.241 | Glutamine, Glucose, Total lipids in medium HDL | 0.007 | 0.056, 0.24, -0.024 |

b)

| **p=0.01** | | | **p=0.05** | | | **p=0.1** | | | **p=0.2** | | |
| --- | --- | --- | --- | --- | --- | --- | --- | --- | --- | --- | --- |
| **Model** | **Marginal inclusion probability (MIP)** | **Model averaged causal effect** | **Model** | **Marginal inclusion probability (MIP)** | **Model averaged causal effect** | **Model** | **Marginal inclusion probability (MIP)** | **Model averaged causal effect** | **Model** | **Marginal inclusion probability (MIP)** | **Model averaged causal effect** |
| Glucose | 1 | 0.243 | Glucose | 1 | 0.241 | Glucose | 1 | 0.239 | Glucose | 1 | 0.237 |
| Glutamine | 0.099 | 0.006 | Glutamine | 0.367 | 0.021 | Glutamine | 0.551 | 0.032 | Glutamine | 0.733 | 0.042 |
| Alanine | 0.011 | 0.001 | Alanine | 0.058 | 0.004 | Alanine | 0.117 | 0.008 | Alanine | 0.232 | 0.016 |
| Lactate | 0.003 | 0 | Lactate | 0.019 | 0.001 | Lactate | 0.041 | 0.003 | Lactate | 0.088 | 0.007 |
| Total lipids in small HDL | 0.002 | 0 | Total lipids in small HDL | 0.01 | 0 | Total lipids in small HDL | 0.021 | -0.001 | Total lipids in small HDL | 0.047 | -0.002 |
| Acetoacetate | 0.001 | 0 | Acetoacetate | 0.006 | 0 | Acetate | 0.014 | -0.001 | Acetate | 0.032 | -0.002 |
| Total lipids in medium HDL | 0.001 | 0 | Acetate | 0.006 | 0 | Total lipids in medium HDL | 0.013 | 0 | Total lipids in medium HDL | 0.029 | -0.001 |
| Acetate | 0.001 | 0 | Total lipids in medium HDL | 0.006 | 0 | Acetoacetate | 0.012 | -0.001 | Acetoacetate | 0.023 | -0.001 |
| Glycine | 0.001 | 0 | Glycine | 0.004 | 0 | Glycine | 0.009 | 0 | Apolipoprotein A1 | 0.02 | 0 |
| Apolipoprotein A1 | 0.001 | 0 | Apolipoprotein A1 | 0.004 | 0 | Apolipoprotein A1 | 0.009 | 0 | Glycine | 0.018 | 0 |

*Table S5a, b: We hold the prior probability constant at default* $(p=0.1)$ *and vary the prior sigma* $\sigma^{2}$*,* $\sigma^{2}=(0.10, 0.30, 0.50, 0.70$*) to check the robustness of results and sensitivity to model selection parameters for the top 10 ranked sets of exposures. The top 10 ranked of sets of exposures for birthweight according to their posterior probability (a) and marginal inclusion probability (b).*

a)

| **𝛔=0.10** | | | **𝛔=0.30** | | | **𝛔=0.50** | | | **𝛔=0.70** | | |
| --- | --- | --- | --- | --- | --- | --- | --- | --- | --- | --- | --- |
| **Model** | **Posterior probability (PP)** | **Model specific causal estimates** | **Model** | **Posterior probability (PP)** | **Model specific causal estimates** | **Model** | **Posterior probability (PP)** | **Model specific causal estimates** | **Model** | **Posterior probability (PP)** | **Model specific causal estimates** |
| Glutamine, Glucose | 0.161 | 0.058, 0.237 | Glutamine, Glucose | 0.36 | 0.058, 0.237 | Glutamine, Glucose | 0.377 | 0.058, 0.237 | Glucose | 0.41 | 0.244 |
| Alanine, Glutamine, Glucose | 0.113 | 0.067, 0.058, 0.221 | Glucose | 0.179 | 0.244 | Glucose | 0.311 | 0.244 | Glutamine, Glucose | 0.355 | 0.058, 0.237 |
| Glutamine, Glucose, Lactate | 0.03 | 0.06, 0.254, 0.076 | Alanine, Glutamine, Glucose | 0.082 | 0.067, 0.058, 0.221 | Alanine, Glutamine, Glucose | 0.051 | 0.067, 0.058, 0.221 | Alanine, Glucose | 0.036 | 0.066, 0.228 |
| Glucose | 0.027 | 0.244 | Alanine, Glucose | 0.036 | 0.066, 0.228 | Alanine, Glucose | 0.038 | 0.066, 0.228 | Alanine, Glutamine, Glucose | 0.034 | 0.067, 0.058, 0.221 |
| Alanine, Glucose | 0.017 | 0.066, 0.228 | Glutamine, Glucose, Lactate | 0.033 | 0.06, 0.254, 0.076 | Glutamine, Glucose, Lactate | 0.021 | 0.06, 0.254, 0.076 | Glutamine, Glucose, Lactate | 0.014 | 0.06, 0.254, 0.076 |
| Alanine, Glutamine, Glucose, Total lipids in small HDL | 0.013 | 0.076, 0.053, 0.229, -0.036 | Acetate, Glutamine, Glucose | 0.011 | -0.057, 0.061, 0.243 | Glucose, Lactate | 0.01 | 0.26, 0.066 | Glucose, Lactate | 0.009 | 0.26, 0.066 |
| Acetate, Glutamine, Glucose | 0.013 | -0.057, 0.061, 0.243 | Glucose, Lactate | 0.009 | 0.26, 0.066 | Glucose, Total lipids in small HDL | 0.007 | 0.254, -0.035 | Glucose, Total lipids in small HDL | 0.007 | 0.254, -0.035 |
| Alanine, Glutamine, Glucose, Total lipids in medium HDL | 0.01 | 0.072, 0.056, 0.223, -0.029 | Glucose, Total lipids in small HDL | 0.007 | 0.254, -0.035 | Acetate, Glutamine, Glucose | 0.007 | -0.057, 0.061, 0.243 | Acetate, Glutamine, Glucose | 0.005 | -0.057, 0.061, 0.243 |
| Acetoacetate, Glutamine, Glucose | 0.008 | -0.04, 0.056, 0.241 | Acetoacetate, Glutamine, Glucose | 0.007 | -0.04, 0.056, 0.241 | Acetoacetate, Glucose | 0.005 | -0.05, 0.249 | Acetoacetate, Glucose | 0.005 | -0.05, 0.249 |
| Glutamine, Glucose, Total lipids in medium HDL | 0.008 | 0.056, 0.24, -0.024 | Glutamine, Glucose, Total lipids in small HDL | 0.006 | 0.054, 0.245, -0.027 | Acetoacetate, Glutamine, Glucose | 0.004 | -0.04, 0.056, 0.241 | Glucose, Total lipids in medium HDL | 0.004 | 0.247, -0.026 |

b)

| **𝛔=0.10** | | | **𝛔=0.30** | | | **𝛔=0.50** | | | **𝛔=0.70** | | |
| --- | --- | --- | --- | --- | --- | --- | --- | --- | --- | --- | --- |
| **Model** | **Marginal inclusion probability (MIP)** | **Model specific causal estimates** | **Model** | **Marginal inclusion probability (MIP)** | **Model specific causal estimates** | **Model** | **Marginal inclusion probability (MIP)** | **Model specific causal estimates** | **Model** | **Marginal inclusion probability (MIP)** | **Model specific causal estimates** |
| Glucose | 1 | 0.226 | Glucose | 1 | 0.237 | Glucose | 1 | 0.239 | Glucose | 1 | 0.24 |
| Glutamine | 0.85 | 0.049 | Glutamine | 0.671 | 0.039 | Glutamine | 0.551 | 0.032 | Glutamine | 0.467 | 0.027 |
| Alanine | 0.417 | 0.028 | Alanine | 0.183 | 0.012 | Alanine | 0.117 | 0.008 | Alanine | 0.085 | 0.006 |
| Lactate | 0.113 | 0.007 | Lactate | 0.066 | 0.005 | Lactate | 0.041 | 0.003 | Lactate | 0.029 | 0.002 |
| Total lipids in small HDL | 0.088 | -0.003 | Total lipids in small HDL | 0.034 | -0.001 | Total lipids in small HDL | 0.021 | -0.001 | Total lipids in small HDL | 0.015 | -0.001 |
| Total lipids in medium HDL | 0.06 | -0.002 | Acetate | 0.023 | -0.001 | Acetate | 0.014 | -0.001 | Acetate | 0.01 | 0 |
| Acetate | 0.054 | -0.002 | Total lipids in medium HDL | 0.021 | -0.001 | Total lipids in medium HDL | 0.013 | 0 | Acetoacetate | 0.009 | 0 |
| Apolipoprotein A1 | 0.044 | -0.001 | Acetoacetate | 0.018 | -0.001 | Acetoacetate | 0.012 | -0.001 | Total lipids in medium HDL | 0.009 | 0 |
| Acetoacetate | 0.039 | -0.001 | Apolipoprotein A1 | 0.015 | 0 | Glycine | 0.009 | 0 | Glycine | 0.006 | 0 |
| Glycine | 0.039 | 0.001 | Glycine | 0.014 | 0 | Apolipoprotein A1 | 0.009 | 0 | Apolipoprotein A1 | 0.006 | 0 |

*Table S6: MVMR-BMA results using the default prior variance* $(\sigma^{2}=0.5)$*, prior probability* $(p=0.1)$ *over 500,000 iterations. The top 10 ranked of sets of exposures for birthweight according to their posterior probability (a) and marginal inclusion probability (b).*

*(a)*

| **Model** | **Posterior probability (PP)** | **Model specific causal estimates** |
| --- | --- | --- |
| Glutamine, Glucose | 0.3765 | 0.0576, 0.23651 |
| Glucose | 0.31077 | 0.2441 |
| Alanine, Glutamine, Glucose | 0.05119 | 0.0665, 0.05779, 0.22074 |
| Alanine, Glucose | 0.0378 | 0.06611, 0.22845 |
| Glutamine, Glucose, Lactate | 0.02129 | 0.06017, 0.25436, 0.07595 |
| Glucose, Lactate | 0.00998 | 0.25997, 0.06628 |
| Glucose, Total lipids in small HDL | 0.00731 | 0.25384, -0.03464 |
| Acetate, Glutamine, Glucose | 0.00729 | -0.05653, 0.06087, 0.24267 |
| Acetoacetate, Glucose | 0.00479 | -0.05016, 0.24888 |
| Acetoacetate, Glutamine, Glucose | 0.00419 | -0.04014, 0.05613, 0.24052 |

*(b)*

| **Exposure** | **Marginal inclusion probability (MIP)** | **Model averaged causal effect** |
| --- | --- | --- |
| Glucose | 1 | 0.23915 |
| Glutamine | 0.55104 | 0.03181 |
| Alanine | 0.11695 | 0.00786 |
| Lactate | 0.04109 | 0.00299 |
| Total lipids in small HDL | 0.021 | -0.00075 |
| Acetate | 0.01418 | -7.00E-04 |
| Total lipids in medium HDL | 0.01262 | -0.00035 |
| Acetoacetate | 0.01212 | -0.00053 |
| Glycine | 0.00876 | 0.00016 |
| Apolipoprotein A1 | 0.00876 | -0.00019 |

*Table S7: The top 30 genetic variants with the largest maximum Q-statistic*

| 1. **UKBB selected SNPs** | |
| --- | --- |
| **Genetic variant** | **Q-statistic** |
| rs7137828 | 42.265 |
| rs2168101 | 24.046 |
| rs1801133 | 23.977 |
| rs1717200 | 17.767 |
| rs76895963 | 15.666 |
| rs4240624 | 15.615 |
| rs113674212 | 14.622 |
| rs34284056 | 13.08 |
| rs117810762 | 11.864 |
| rs61907563 | 11.355 |
| rs79687284 | 11.158 |
| rs472031 | 11.045 |
| rs9878347 | 11.022 |
| rs6592965 | 10.335 |
| rs560887 | 8.741 |
| rs9492442 | 8.458 |
| rs11720108 | 8.186 |
| rs4237150 | 8.14 |
| rs11086986 | 8.069 |
| rs4946935 | 7.714 |
| rs2271386 | 7.308 |
| rs17122673 | 7.291 |
| rs36181536 | 7.197 |
| rs56335308 | 7.146 |
| rs1900349 | 6.958 |
| rs150844304 | 6.763 |
| rs561931 | 6.422 |
| rs62182473 | 6.14 |
| rs76837685 | 5.959 |
| rs4948102 | 5.948 |

*Table S8: The top 30 genetic variants with the largest maximum Cooks distance for the best model.*

| 1. **UKBB selected SNPs** | |
| --- | --- |
| **Genetic variant** | **Cook’s D** |
| rs7137828 | 0.101 |
| rs6065904 | 0.079 |
| rs4240624 | 0.062 |
| rs560887 | 0.062 |
| rs117810762 | 0.045 |
| rs2168101 | 0.035 |
| rs113674212 | 0.031 |
| rs58542926 | 0.028 |
| rs1260326 | 0.028 |
| rs150844304 | 0.026 |
| rs2388595 | 0.025 |
| rs10018448 | 0.022 |
| rs11745373 | 0.022 |
| rs1047891 | 0.022 |
| rs79687284 | 0.02 |
| rs72786786 | 0.018 |
| rs118147862 | 0.016 |
| rs11902417 | 0.013 |
| rs1801133 | 0.012 |
| rs4846921 | 0.012 |
| rs429358 | 0.012 |
| rs71386944 | 0.012 |
| rs1077835 | 0.011 |
| rs17122673 | 0.011 |
| rs2939302 | 0.011 |
| rs472031 | 0.011 |
| rs76895963 | 0.01 |
| rs9878347 | 0.01 |
| rs1601935 | 0.01 |
| rs72789541 | 0.01 |

*Table S9: The number of SNPs associated with each metabolite in the main analysis.*

| **Metabolite** | **SNPs (N)** | **Metabolite** | **SNPs (N)** |
| --- | --- | --- | --- |
| Total triglycerides | 48 | Total lipids in medium HDL | 46 |
| Acetate | 5 | Total lipids in medium LDL | 33 |
| Acetoacetate | 4 | Total lipids in medium VLDL | 44 |
| Acetone | 5 | Monounsaturated fatty acids | 42 |
| Alanine | 17 | Omega-3 fatty acids | 46 |
| Albumin | 22 | Omega-6 fatty acids | 30 |
| Apolipoprotein A1 | 50 | Phenylalanine | 9 |
| Apolipoprotein B | 38 | Phosphatidylcholines | 37 |
| 3-Hydroxybutyrate | 5 | Pyruvate | 16 |
| Citrate | 25 | Total lipids in small HDL | 26 |
| Creatinine | 54 | Total lipids in small VLDL | 45 |
| Docosahexaenoic acid | 33 | Saturated fatty acids | 34 |
| Glutamine | 38 | Sphingomyelins | 26 |
| Glucose | 17 | Total esterified cholesterol | 33 |
| Glycine | 52 | Total free cholesterol | 32 |
| Glycoprotein acetyls | 42 | Total phospholipids in lipoprotein particles | 34 |
| Histidine | 16 | Tyrosine | 22 |
| Total lipids in IDL | 36 | Valine | 12 |
| Isoleucine | 5 | Total lipids in very large HDL | 72 |
| Total lipids in large HDL | 86 | Total lipids in very small VLDL | 50 |
| Total lipids in large LDL | 38 | Total lipids in chylomicrons and extremely large VLDL | 53 |
| Total lipids in large VLDL | 44 |  |  |
| Linoleic acid | 28 |  |  |
| Lactate | 7 |  |  |
| Leucine | 9 |  |  |

*Table S10: Calculated F-statistics or conditional F-statistics for maternal metabolites on birthweight in the primary analysis.*

| **Model** | **F-statistic^1^** | **F-statistic using all SNPs** |
| --- | --- | --- |
| Glucose, Glutamine | 83.603, 26.632 | 4.805, 12.207 |
| Glucose | 70.963 | 4.759 |
| Alanine, Glutamine, Glucose | 27.135, 74.107, 21.118 | 5.288, 12.528, 4.637 |
| Alanine, Glucose | 40.182, 31.716 | 5.152, 4.637 |
| Glutamine, Glucose, Lactate | 69.115, 26.403, 8.787 | 12.009, 4.916, 2.751 |
| Glucose, Lactate | 55.972, 18.246 | 4.865, 2.768 |
| Glucose, Total lipids in small HDL | 22.628, 59.002 | 4.796, 11.596 |
| Acetate, Glutamine, Glucose | 9.226, 79.175, 24.560 | 2.375, 12.275, 4.820 |
| Acetoacetate, Glucose | 16.670, 53.348 | 2.630, 4.963 |
| Acetoacetate, Glutamine, Glucose | 8.376, 68.709, 24.680 | 2.616, 12.087, 4.987 |

*^1^ The mean F-statistic was calculated for univariable models and the conditional F-statistic for multivariable models.*

*Table S11: MVMR-BMA results from sensitivity analysis of exposure inclusion using the default prior variance* $(\sigma^{2}=0.5)$*, prior probability* $(p=0.1)$ *over 10,000 iterations. The top 10 ranked of sets of exposures for birthweight according to their posterior probability (a) and marginal inclusion probability (b).*

*(a)*

| **Model** | **Posterior probability (PP)** | **Model specific causal estimates** |
| --- | --- | --- |
| Glutamine,Glucose | 0.37742 | 0.0576,0.23651 |
| Glucose | 0.31152 | 0.2441 |
| Alanine,Glutamine,Glucose | 0.05131 | 0.0665,0.05779,0.22074 |
| Alanine,Glucose | 0.03789 | 0.06611,0.22845 |
| Glutamine,Glucose,Lactate | 0.02134 | 0.06017,0.25436,0.07595 |
| Glucose,Lactate | 0.01 | 0.25997,0.06628 |
| Glucose,Total lipids in small HDL | 0.00733 | 0.25384,-0.03464 |
| Acetate,Glutamine,Glucose | 0.0073 | -0.05653,0.06087,0.24267 |
| Acetoacetate,Glucose | 0.00481 | -0.05016,0.24888 |
| Acetoacetate,Glutamine,Glucose | 0.0042 | -0.04014,0.05613,0.24052 |

*(b)*

| **Exposure** | **Marginal inclusion probability (MIP)** | **Model averaged causal effect** |
| --- | --- | --- |
| Glucose | 1 | 0.23914 |
| Glutamine | 0.55099 | 0.03181 |
| Alanine | 0.11619 | 0.00781 |
| Lactate | 0.04014 | 0.00292 |
| Total lipids in small HDL | 0.02058 | -0.00073 |
| Acetate | 0.01402 | -0.00069 |
| Total lipids in medium HDL | 0.01244 | -0.00034 |
| Acetoacetate | 0.01189 | -0.00053 |
| Glycine | 0.00869 | 0.00016 |
| Apolipoprotein A1 | 0.00861 | -0.00019 |

**Supplementary Figures**

**
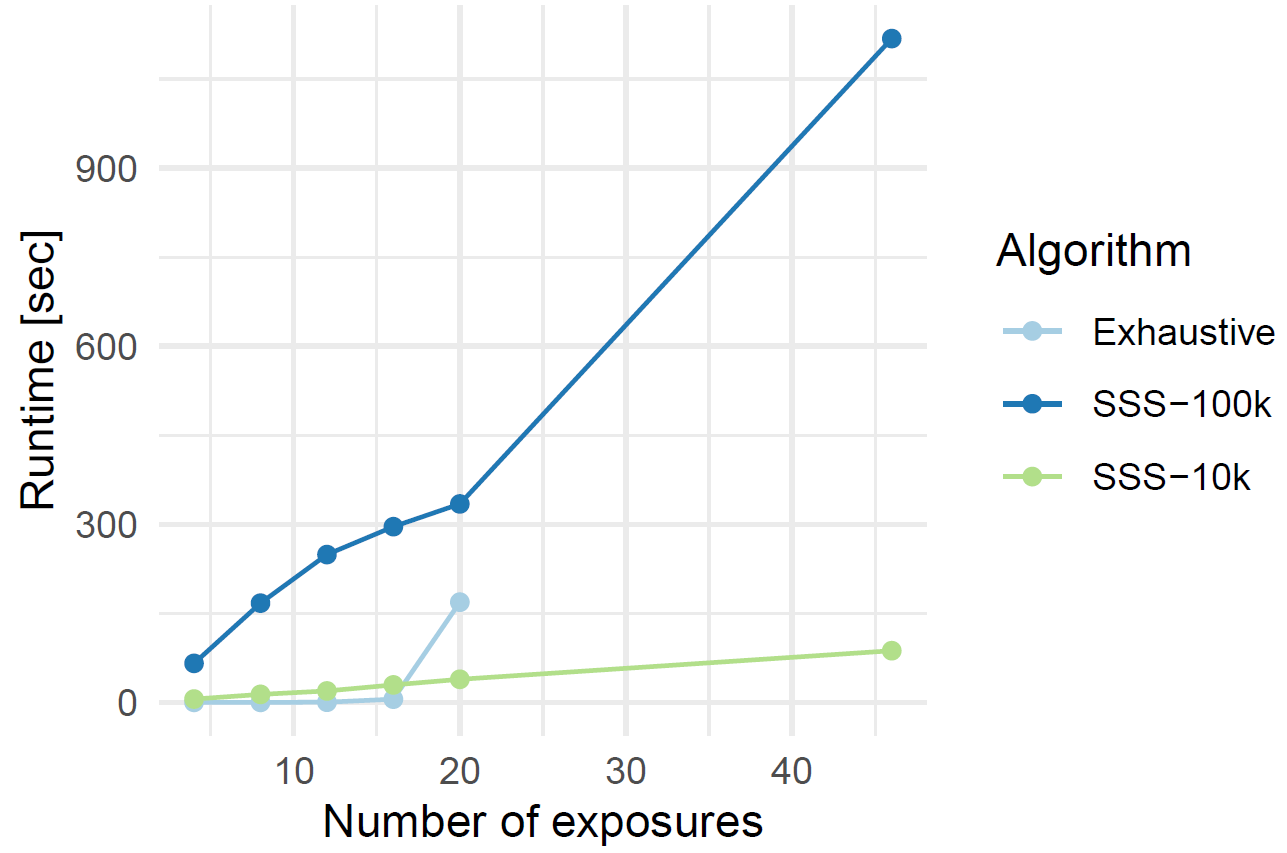
**

Figure S1: Plot of runtime (in seconds) against number of exposures. SSS: shotgun stochastic search, 10K: 10,000 iterations and 100K: 100,000 iterations.

a)


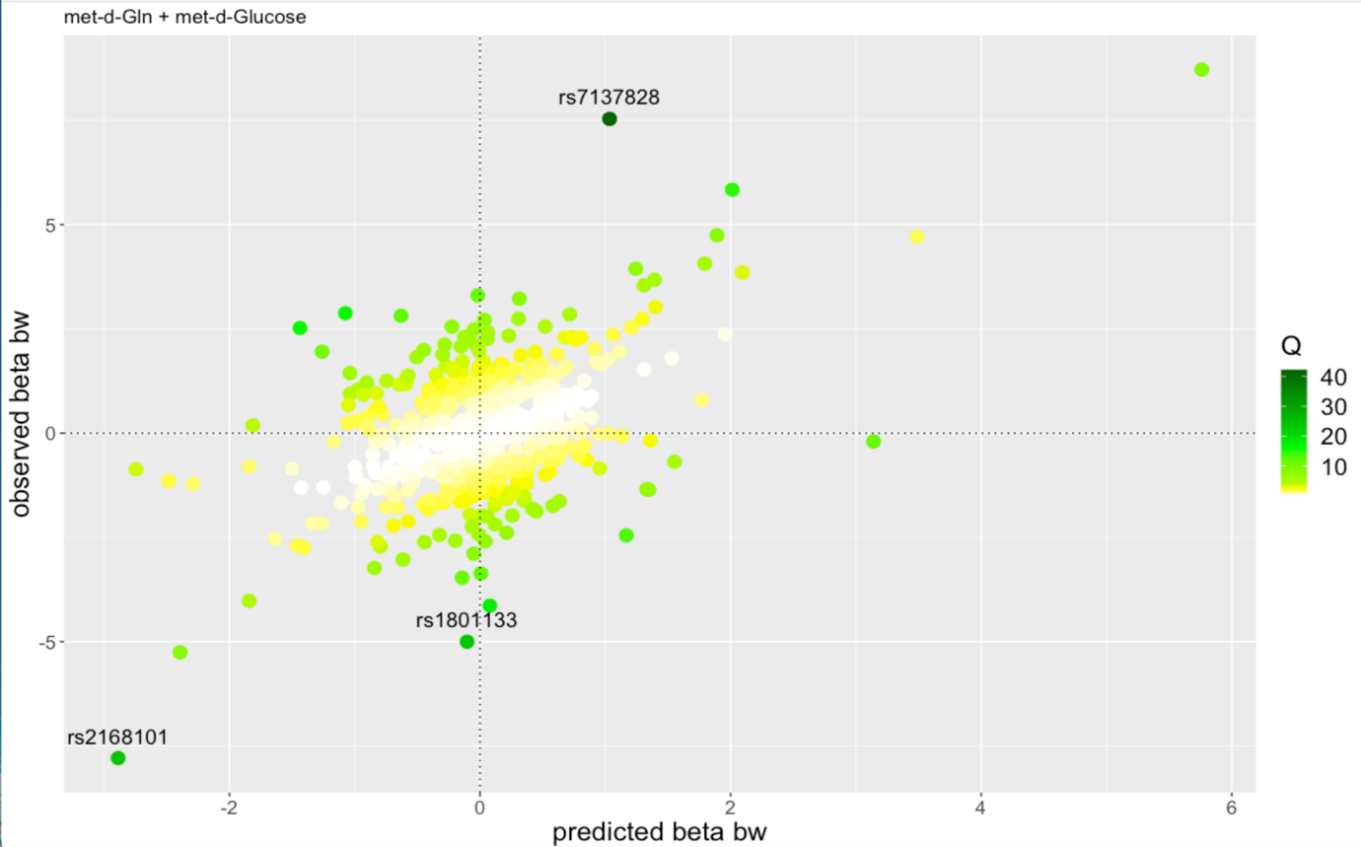


b)


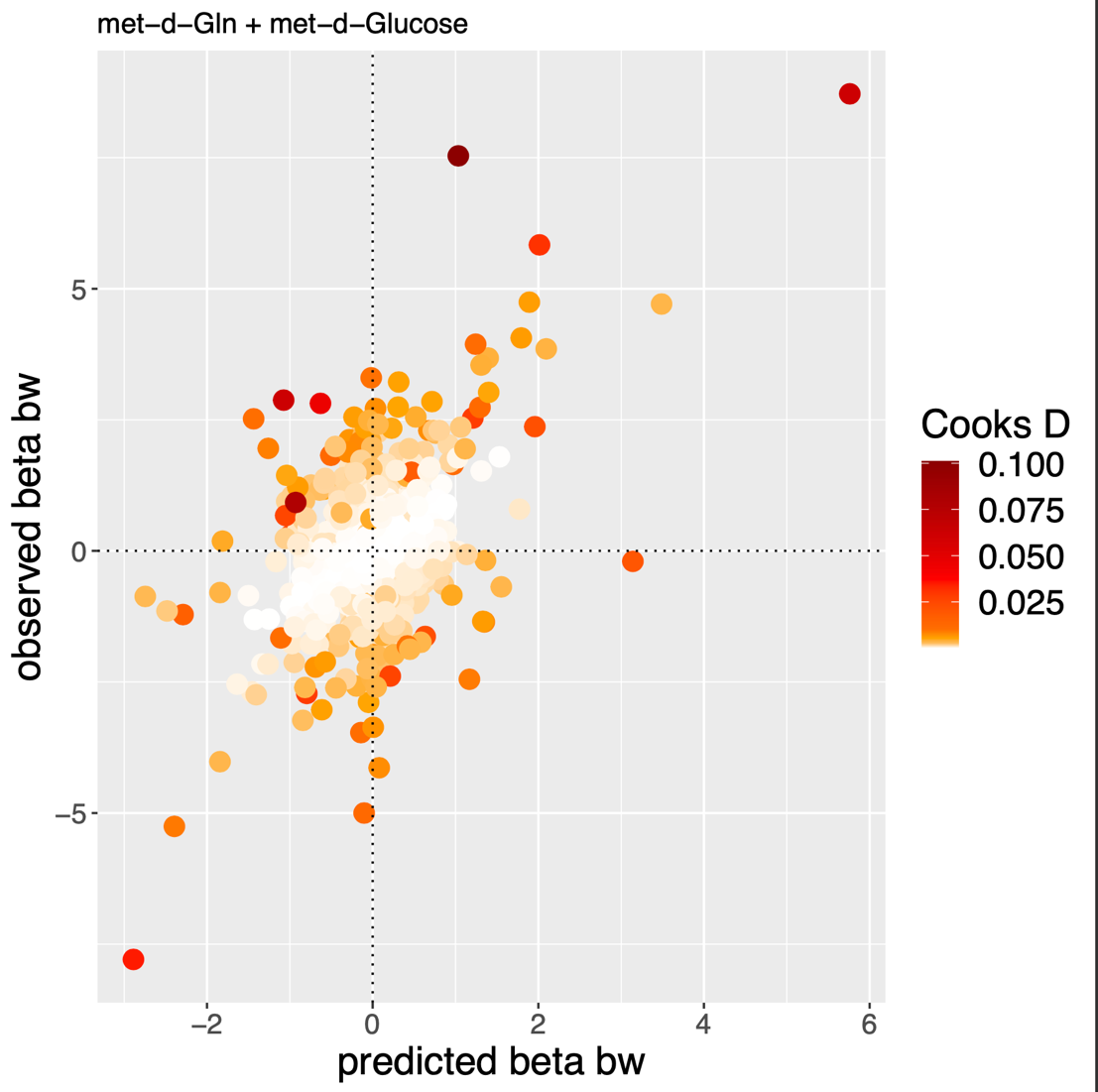


Figure S2a, b: *Diagnostic plots for the highest ranked model (glutamine and glucose) selected using default prior variance and probability for UKBB selected SNPs. Figure a, the q-statistic of the SNPs, indicating outliers, and Figure b the Cook’s distance for influential points. SNPs exceeding the Cook’s distance threshold are marked by their SNP rs number.*

**References**

1. Zuber, V., et al., *Selecting likely causal risk factors from high-throughput experiments using multivariable Mendelian randomization.* Nature Communications, 2020. **11**(1): p. 29.

2. Hemani, G., et al., *The MR-Base platform supports systematic causal inference across the human phenome.* eLife, 2018. **7**: p. e34408.

3. Elsworth, B., et al., *The MRC IEU OpenGWAS data infrastructure.* bioRxiv, 2020: p. 2020.08.10.244293.

4. Bycroft, C., et al., *The UK Biobank resource with deep phenotyping and genomic data.* Nature, 2018. **562**(7726): p. 203-209.

5. Elsworth, B., et al., *MRC IEU UK Biobank GWAS pipeline version 2.* University of Bristol, 2018. **10**.

6. Sanderson, E. and F. Windmeijer, *A weak instrument F-test in linear IV models with multiple endogenous variables.* Journal of Econometrics, 2016. **190**(2): p. 212-221.

7. Sanderson, E., W. Spiller, and J. Bowden, *Testing and correcting for weak and pleiotropic instruments in two-sample multivariable mendelian randomisation.* Statistics in medicine, 2021.

8. Brereton, R.G., *Empirical and statistical p values and Type 1 error rates: Putting it all together.* Journal of Chemometrics, 2021. **35**(9): p. e3330.
